# Gait Analysis for Identifying Normal Cognition, Subjective Cognitive Decline, and Mild Cognitive Impairment in Parkinson Disease: Diagnostic Study

**DOI:** 10.2196/69273

**Published:** 2026-06-24

**Authors:** Juan Huang, Lingyu Wu, Hui Wang, Lin Chen, Binbin Hu, Fei Zhang, Kang Ren, Yun Ling, Zhonglue Chen, Wei Huang

**Affiliations:** 1 Department of Neurology Jiangxi Medical College, Nanchang University The Second Affiliated Hospital of Nanchang University Nanchang, Jiangxi China; 2 Department of Neurology The First affiliated Hospital of Nanhua University Hengyang China; 3 GYENNO Science Co., Ltd. Shenzhen China; 4 HUST-GYENNO CNS Intelligent Digital Medicine Technology Center School of Artificial Intelligence and Automation Huazhong University of Science and Technology Wuhan, Hubei China; 5 Guangdong Provincial Engineering Technology Research Center for Medical Artificial Intelligence in Neurological Disease Shenzhen, Guangdong China

**Keywords:** Parkinson disease, subjective cognitive decline, mild cognitive impairment, wearable sensor, kinematic parameters, machine learning

## Abstract

**Background:**

Patients with Parkinson disease (PD) along with subjective cognitive decline (PD-SCD) are considered an intermediate status between those with normal cognition (PD-NC) and those with mild cognitive impairment (PD-MCI). Wearable digital monitoring technologies and machine learning models offer significant potential for assessing cognitive impairment in patients with PD.

**Objective:**

We aimed to evaluate the utility of wearable technology and machine learning for identifying ordinal cognitive stages (OCS) in PD based on timed up and go (TUG) tests (including single-task TUG [TUGst] and dual-task TUG [TUGdt]).

**Methods:**

Patients with PD along with SCD, MCI, and NC were recruited in a movement disorder clinic. Patients performed TUGst and TUGdt gait trials wearing a motor function and motor symptom quantitative assessment system. In total, 209 kinematic parameters were synthesized for individual TUG to illustrate patients’ motion profiles. We constructed dual-task cost parameters (DTC), describing the magnitude of the effect of the cognitive challenge on motion performance. Covariate-adjusted ordered logistic regression was used to compare parameter differences among 3 groups. Multiple machine learning models were used to classify the participants into 3 cognitive impairment levels, with features being selected based on *P* values from intergroup statistical tests. The total population was randomly divided into a training set and an independent validation set in a 7:3 ratio, and 10-fold cross-validation was used in the training set. Furthermore, this study used permutation importance and Shapley Additive Explanations (SHAP) analysis (including summary plots, bar plots, and waterfall plots) to explain the feature importance of the final model.

**Results:**

The study included 65 age-matched patients (PD-NC: PD-SCD: PD-MCI= 14:21:30). Forty-five kinematic parameters were significantly different (*P*<.05) among the 3 groups, distributed across TUGst (n=25), TUGdt (n=12), and DTC (n=8) paradigms. Gait phase analysis revealed 35 parameters from walking phases, 9 from stand-to-sit transitions, and 1 from sit-to-stand transitions. Feature type distribution demonstrated predominance of variability features (n=20), followed by pace (n=12) and axial (n=8) characteristics. TUGdt paradigm analysis revealed pronounced movement differences between PD-MCI and both PD-NC and PD-SCD groups, particularly in variability, amplitude, pace, and axial domains. Cross-paradigm analysis identified consistent significant differences in specific features. These findings provide objective kinematic biomarkers for early cognitive state identification in Parkinson disease, with TUGdt parameters demonstrating superior discriminative capacity.

**Conclusions:**

This suggests patients with PD-SCD could have early kinetic signs of cognitive impairment, positioning them between PD-NC and PD-MCI, and our multiclass support vector machine classification model with kinematic parameters achieved a recall rate above 0.70 in both training and validation datasets. The feature importance analysis revealed that DTC_Trunk-Right Rotation Max, DTC_Trunk-Max Transverse Angular Velocity, and dTUG_Lumbar-Right Sway Max Std were the most critical features for distinguishing cognitive states, providing scientific evidence for cognitive function screening based on kinematic parameters.

## Introduction

Parkinson disease (PD) with subjective cognitive decline (SCD) [1] is significantly associated with cognitive impairment, including attention and executive function abilities [2]. At the time of this writing, it is suggested that the cognition performance of patients with PD can be classified into three stages: (1) SCD as the prodromal phase (PD-SCD), (2) followed by MCI (PD-MCI), and (3) finally leading to dementia [3-6]. Longitudinal studies revealed that PD-SCD is more likely to develop into PD-MCI, and PD-SCD may have the potential to predict cognitive decline later [7]. Thus, it is essential to identify individuals with PD-SCD as early as possible.

Brain imaging showed functional and structural correlates between motor control and cognition [8]. Evidence implicates dopamine-dependent basal ganglia dysfunction in PD-related dual-task gait impairments. As the basal ganglia regulate automatic motor control, their degeneration may hinder concurrent motor-cognitive processing during walking [9]. In PD, basal ganglia dysfunction reduces movement automaticity, forcing increased cognitive control of actions. The functional magnetic resonance imaging [10] shows patients with PD overrecruit the premotor and prefrontal cortices during dual-task arm movements versus controls, a pattern also observed in single-task walking, suggesting pervasive compensatory cognitive engagement [11]. Notably, increased quantitative susceptibility mapping values in the cuneus significantly correlated with poorer Montreal Cognitive Assessment (MoCA) performance. The patients with PD-MCI showed elevated quantitative susceptibility mapping values in the cuneus, precuneus, caudate head, fusiform gyrus, and orbitofrontal cortex relative to cognitively normal PD controls [12-14]. Collectively, PD-related dopaminergic degeneration concurrently impairs motor and cognitive circuits, with particular dysfunction in the basal ganglia [15].

Timed Up and Go (TUG) tests (eg, single-task TUG [TUGst] tests and dual-task cognition-gait TUG [TUGdt] tests) have been commonly used to assess motor performance and illustrate the association between motor performance and cognition. The TUGdt test reflects motor-cognitive interactions by challenging the cognitive component of locomotion and thus can provide insight into the mechanisms underlying brain motor control and cognitive performance [16,17]. Gait modifications (ie, dual-task costs [DTCs]) are interpreted to reflect the magnitude of changes in motor performance when cortical attention is involved in gait processes [17].

Wearable digital monitoring technologies combined with machine learning models potentially have considerable value for patients with PD and cognitive impairment [18,9]. Previous studies have demonstrated that PD-MCI exhibits greater postural instability and gait disturbances compared to those without MCI (PD-noMCI), particularly under dual-task conditions [19,20]. Russo et al [21,22], further corroborated the strong association between gait dysfunction and MCI in PD, reporting that patients with PD-MCI present with cognitive decline alongside more severe axial motor symptoms and increased postural flexion. Additionally, meta-analyses by other researchers have underscored the diagnostic value of integrating Kinect-based gait analysis—a critical behavioral marker—with machine learning for identifying cognitive impairment in PD [23,24].

The diagnostic criteria for PD-SCD are mainly based on subjective scales [25-27], and the variety of very diverse instruments used to identify SCD could potentially introduce bias. Thus, research on SCD has predominantly focused on areas such as assessment questionnaires, neuroimaging, and associations with emotion and/or objective cognitive functions [28,29]. There have been few studies combining wearable devices and machine learning methodologies for early screening. This gap is important because larger longitudinal studies have shown that patients with Parkinson disease—subjective cognitive decline have a higher likelihood of progressing to Parkinson disease—mild cognitive impairment within a 3-year follow-up period, highlighting the predictive value of PD-SCD for subsequent cognitive decline [7].

In this study, we hypothesized that patients with PD-SCD were intermediate in cognitive status between patients with PD with normal cognition (PD-NC) and PD-MCI. This study aimed to evaluate the utility of wearable technology and machine learning for identifying ordinal cognitive stages (OCS) in patients with PD based on TUG tests (eg, single-task TUG [TUGst] tests and dual-task cognition-gait TUG [TUGdt] tests), which have been commonly used to assess motor performance and demonstrate the association between motor performance and cognition [17]. To our knowledge, this study is pioneering in its inclusion of individuals with PD-SCD within a framework that uses wearable sensors and machine learning for screening.

## Methods

### Recruitment

**Figure 1:**
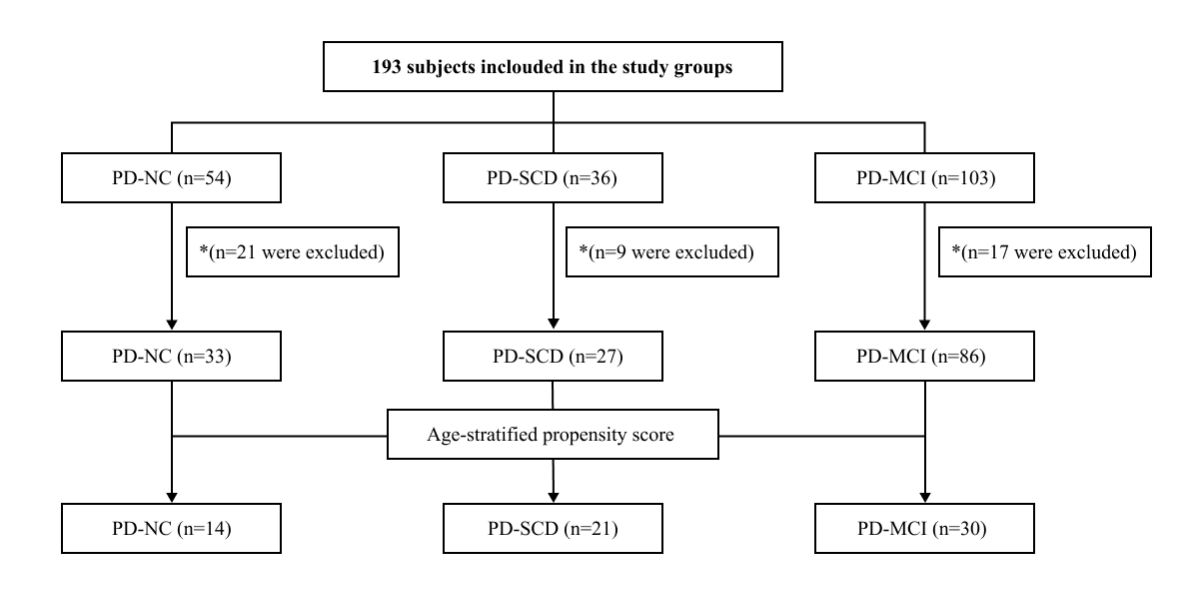
The data screening flowchart. PD-NC: Parkinson disease-normal cognition; PD-SCD: Parkinson disease-subjective cognitive decline; *The data are excluded because of the incomplete demographic records, partial scale completion, or the incomplete gait measurement. PD-MCI: Parkinson disease-mild cognitive impairment;.

The data collection process in this study used a rigorous screening and matching strategy (Figure 1). The initial research cohort comprised 193 participants who were preliminarily categorized into 3 groups based on cognitive functional status: PD with normal cognition (PD-NC; n=54), PD with subjective cognitive decline (PD-SCD; n=36), and PD with mild cognitive impairment (PD-MCI; n=103). During the first-stage screening process, some participants were excluded due to incomplete demographic records, partial scale completion, or missing gait measurement data. Specifically, 21 cases were excluded from the PD-NC group, 9 cases from the PD-SCD group, and 17 cases from the PD-MCI group. After preliminary screening, the sample sizes for each group were 33 cases for PD-NC, 27 cases for PD-SCD, and 86 cases for PD-MCI. To control for the influence of age as a significant confounding factor on research outcomes, we used age-stratified propensity score matching for all participants. This matching process ensured balanced age distribution across the 3 groups, effectively controlling for age differences’ impact on cognitive function and gait performance. After age matching, the final sample size included in the analysis was 65 participants, consisting of 14 cases in the PD-NC group, 21 cases in the PD-SCD group, and 30 cases in the PD-MCI group. This matching strategy not only enhanced the internal validity of the study but also provided a more reliable data foundation for subsequent machine learning model analysis.

A consecutive inclusion of 65 age-matched patients from a movement disorder clinic was undertaken (PD-NC [n=14]; PD-SCD [n=21]; and PD-MCI [n=30]). Both physical and thorough interviews were done with them. Age, gender, education level, and duration of disease were recorded. All of the patients were diagnosed with idiopathic PD according to the International Parkinson and Movement Disorder Society (MDS) clinical diagnostic criteria. The inclusion criteria were as follows: (1) diagnosis of PD according to the criteria of the MDS, (2) stages 1-3 on the Hoehn-Yahr (H-Y) scale, (3) stable use of antiparkinsonian medication based on medical history, and (4) ability to walk independently for at least 5 minutes. Patients were excluded based on the following criteria: (1) those who diagnosed dementia, (2) a diagnose of secondary PD, (3) medical history indicates having history of brain disorders (eg, cerebral infarction, cerebral hemorrhage, or brain tumor), and (4) those with serious psychiatric diseases (eg, Hamilton Anxiety Scale [HAMA] ≥21 [30], Hamilton Depression Scale [HAMD] ≥17 [31], or obsessive-compulsive disorder). This study was approved by the Research Ethics Committee of the Second Affiliated Hospital of Nanchang University.

### Diagnostic Criteria for PD-NC, PD-SCD, and PD-MCI

The participants underwent a comprehensive neurocognitive assessment. Motor symptom severity was evaluated using the MDS Unified Parkinson Disease Rating Scale (MDS-UPDRS) and H-Y stage [32]. Cognitive function was assessed using the MoCA. The MoCA covers multiple cognitive domains, including memory, language, complex visuospatial processing, and executive function, and has a maximum score of 30. Emotional features were assessed with the HAMA and HAMD, which has been validated for use with patients with PD.

According to the guidelines for the diagnosis of dementia in PD published by the MDS, a cut-off score of <26 is recommended for PD-MCI; additionally, 1 point is added to the total score (if less than 30) for participants with ≤12 years of education [33]. In this study, we define normal global cognition as MoCA ≥25/30 for participants with ≤12 years of education and 26/30 for participants with >12 years of education. Participants answered item 1.1 of MDS-UPDRS Part I, and based on the score, the participants were divided into 2 groups: a score of ≥1 indicated those with subjective cognitive complaints and a score of <1 indicated those without subjective cognitive complaints. Thus, we defined PD-SCD as individuals with item 1.1 of MDS-UPDRS Part I ≥1 as well as normal objective global cognition; PD-NC was identified as patients with PD with item 1.1 of MDS-UPDRS Part I <1 and normal objective global cognition [34,35].

### Single-Task TUG (TUGst) and Dual-Task TUG (TUGdt) Gait Tests

Participants performed single-task TUG (TUGst) and dual-task TUG (TUGdt) gait trials wearing a Motor Function and Motor Symptom Quantitative Assessment System (MATRIX; GYENNO SCIENCE). The MATRIX consists of 10 inertial sensors (ie, 10 data recording channels) sampling at 100 Hz. Each inertial sensor consists of a (1) triaxial accelerometer with range=±16 g and sensitivity=16,384 LSB/g and a (2) triaxial gyroscope with range=±2000 dps and sensitivity=131 LSB/dps. The chest sensor was placed on the sternum of the chest, and the lumbar sensor was attached to the fifth lumbar vertebra. Two wrist sensors were bilaterally placed on the dorsal side of the wrist. Two thigh sensors were bilaterally placed 7 cm above the knee, while 2 shank sensors were bilaterally placed 7 cm below the knee. Two-foot sensors were bilaterally placed at the instep (dorsal side of the metatarsus) of each foot. All sensors were tightened to designated locations using straps. As a 3-axis accelerometer and a 3-axis gyro are combined in one sensor, each sensor of the MATRIX records 6 separate signals synchronously. Therefore, 60 signals (10 sensors×6 signals) were recorded in computers for feature extraction of a single TUG trial per participant. Please refer to previous research literature for the specific attached position and detailed metadata of sensors [36,37].

During the TUGst test, without the use of any mobility aids, participants stood up from a chair, walked 5 meters in a straight line, turned 180°, walked straight back to the chair, turned another 180°, and sat on the chair at their usual pace. During the TUGdt test, participants performed the usual-pace TUG test while subtracting 7 serially from 100 (ie, 93, 86, 79, and so on). To avoid learning effects and fatigue, participants performed both tests quietly and completed only one trial of each test.

### Kinematic Parameters

To illustrate motion profiles of the arms, lumbar spine, trunk, feet, and shanks, 209 kinematic parameters were synthesized for individual TUG trials by gait event recognition based on signal data stored in computers [36,37]. Parameters were listed in Multimedia Appendix 1. This process involved several key aspects: data acquisition and preprocessing using MATRIX with 10 inertial sensors, each recording 6 independent signals (triaxial accelerometer and gyroscope), resulting in 60 raw signals per TUG trial; gait event recognition through algorithmic identification of key gait events, dividing the trial into walking phase, stand-to-sit transition, sit-to-stand transition, and turning phases; parameter extraction and classification based on gait event recognition, extracting 209 kinematic parameters from raw signals covering spatial and temporal characteristics such as velocity, range of motion, stride length, cadence, swing, and stance, as well as asymmetry features including velocity asymmetry and range of motion (RoM) asymmetry; and anatomical plane analysis considering sagittal, coronal, and transverse planes across multiple body segments including arms, lumbar spine, trunk, feet, and shanks. These parameters represent motion asymmetry for bilateral limbs, kinematic variability (SD of parameters), and task-related spatial and temporal characteristics, including velocity (degree per sec), range of motion (RoM, degree), peak velocity (degree per sec), and RoM (degree) of 3 anatomical planes, respectively (parameters the same as lumbar), stride velocity (m/s), cadence (step per min), gait cycle (sec), stride length (cm), step length (cm), swing (%), stance (%), peak angular velocity (degree per sec), RoM (degree), velocity asymmetry (%), RoM asymmetry (%), absolute deviation between limbs, walk duration (sec), and turning duration (sec). According to parameter definitions, 209 parameters were categorized into variability features, amplitude features, pace features, speed features, axial features, and asymmetry features of motion, ensuring comprehensive capture of motion characteristics across all body segments during TUG tasks and providing rich kinematic information for cognitive state classification. Please refer to previous research literature for specific algorithms [37].

Furthermore, DTC parameters, describing the magnitude of the effect of the cognitive challenge on motion performance, were calculated with equation


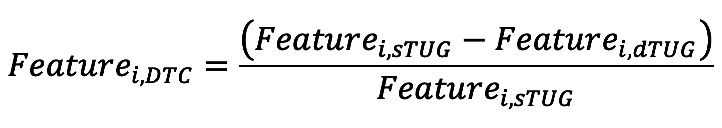
 , in which i refers to each of the 209 parameters.

### Data Analysis

#### Statistics

Quantitative variables following a normal distribution were represented with mean and SD (μ+σ), and statistical differences among multiple groups were tested with one-way ANOVA. Pairwise comparisons between group levels used a student *t* test. Nonnormal quantitative and qualitative or ordinal variables were summarized with median and IQR (lower quartile part or 25th percentile-upper quartile part or 75th percentile) and number of samples and population percentage n (%), respectively. The Kruskal-Wallis rank sum test was used to compare univariate differences on demographic, clinic characteristics, and kinematic parameters among 3 groups. A pairwise Wilcoxon rank sum test was used for 2×2 multiple comparisons between group levels. The ordered logistic regression with covariates (ie, education, age, length of the calf, weight, and BMI) adjusted was used to compare differences on kinematic parameters among multiple groups. Hypothesis testing for categorical variables was performed with the Pearson chi-square test or Fisher exact test. Statistical analyses were conducted using R (version 4.1.0; R Foundation for Statistical Computing) with R Studio (version 1.4.1717; RStudio, PBC).

#### Feature Selection, Modeling, and Interpretability

Kinematic parameters that statistically significantly different among 3 groups (with covariates adjusted in ordered logistic regression) were selected as candidate predictors, resulting in 45 kinematic features. Then, for candidate predictors, 2×2 multiple comparisons were applied to multiply compare the differences between PD-MCI and PD-SCD, PD-MCI and PD-NC, or PD-NC and PD-SCD, respectively. Parameters that significantly different among one of three comparisons were selected as predictors. To optimize model performance and prevent overfitting given the limited sample size (n=65), we selected the top 10 most discriminative features based on their statistical significance and clinical relevance, resulting in a final set of 10 kinematic features as predictors. This feature selection strategy was chosen to balance model complexity with available data, as including too many features relative to sample size can lead to overfitting and reduced generalization ability, while too few features may not capture sufficient discriminative information for accurate classification.

The total population was randomly divided into a training set and an independent validation set in a 7:3 ratio, and random forest, support vector machine (SVM), and K-nearest neighbors algorithms were used to classify the participants into 3 cognitive impairment levels (ie, PD-MCI, PD-SCD, and PD-NC). The training set was used to train and evaluate model performance, and the independent test set was used to test the final model. Ten-fold cross-validation was used to evaluate the classification performance of these models in the training set. Recall, precision, and accuracy were used to measure the accuracy and calculated as follows:

True positive = the number of cases correctly classified as PD-MCI, PD-SCD, or PD-NC;

False positive = the number of cases incorrectly classified as PD-MCI, PD-SCD, or PD-NC;

True negative = the number of cases correctly classified as PD-MCI, PD-SCD, or PD-NC;

False negative = number of cases incorrectly classified as PD-MCI, PD-SCD, or PD-NC;

(1.1) 
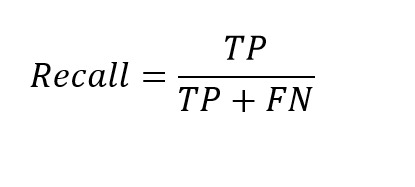


(1.2) 
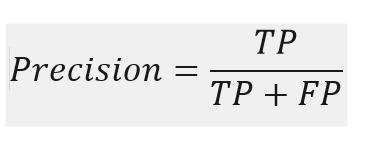


(1.3) 
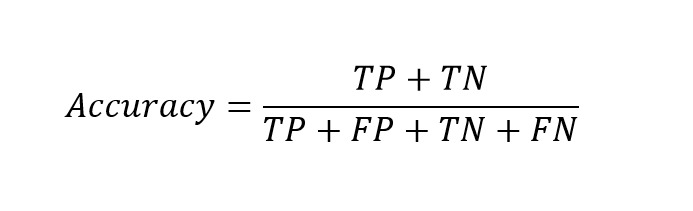


Due to the small sample size (n=65) after age-matching, the model performance might be unstable with repeated data splits. Therefore, the additional validation using the bootstrap method to calculate CIs for recall, precision, and accuracy to evaluate the reliability of model performance.

Furthermore, we used the permutation importance by randomly shuffling the values of a feature and observing the resulting degradation in model performance and mean absolute Shapley Additive Explanations (SHAP) value [38] (mean [|SHAP|]) to measure feature importance. SHAP deconstructs a prediction into a sum of contributions from each of the model’s input variables, thus explaining how individual predictions are made by a machine learning model. And the waterfall plot based on SHAP was employed to visually display how the SHAP values (evidence) of each feature move the model output from our prior expectation under the background data distribution to the final model prediction given the evidence of all the features. The bottom of a waterfall plot starts as the expected value of the model output, and then each row shows how the positive (red) or negative (blue) contribution of each feature moves the value from the expected model output over the background dataset to the model output for this prediction. Mean (|SHAP|) is, on average, how much each variable impacts the predicted probability of participants being PD-NC, PD-MCI, and PD-SCD. The mean absolute SHAP value is a metric that quantifies the average absolute magnitude of SHAP values across all features in a model. Machine learning was conducted using Python (version 3.8.1627; Python Software Foundation) [39].

### Ethical Considerations

This study was approved by the Research Ethics Committee of the Second Affiliated Hospital of Nanchang University, with all participants providing informed consent. Data were anonymized using ID numbers, and follow-up participants received travel compensation.

## Results

### Demographic and Clinic Characteristics in Groups of PD-NC, PD-SCD, and PD-MCI

There were no statistically significant differences among the PD-NC, PD-SCD, and PD-MCI groups in terms of demographic characteristics (including age, sex, height, thigh length, weight, education level, disease duration, and H-Y stage) or scores on MDS-UPDRS I, MDS-UPDRS II, MDS-UPDRS III, HAMA, and HAMD (*P*>.05).

**Figure 2:**
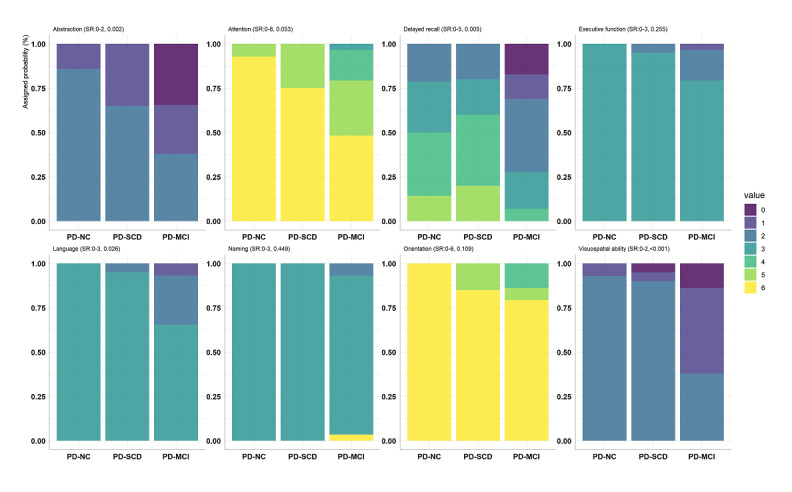
Distribution of Montreal Cognitive Assessment (MoCA) subdomain scores among Parkinson disease-normal cognition (PD-NC), Parkinson disease-subjective cognitive decline (PD-SCD), and Parkinson disease-mild cognitive impairment (PD-MCI). PD-MCI: Parkinson disease-mild cognitive impairment; PD-NC: Parkinson disease-normal cognition; PD-SCD: Parkinson disease-subjective cognitive decline.

The diagnostic criteria for PD-NC, PD-SCD, and PD-MCI were based on UPDRS1.1 scores and MoCA total scores, which showed statistically significant intergroup differences (*P*<.001; Multimedia Appendix 2). The MoCA scale and its subdomain scores exhibited different patterns of differences among the 3 groups (Figure 2). In terms of MoCA total scores, the PD-MCI group (23.00, 95% CI 22.00-25.00) was significantly lower than both the PD-NC group (28.00, 95% CI 27.00-29.00) and the PD-SCD group (27.00, 95% CI 27.00-28.00), while no significant difference was observed between PD-NC and PD-SCD groups. Among the MoCA subdomain scores, statistically significant differences were found among the 3 groups in abstraction (*P*=.002), delayed recall (*P*=.005), and visuospatial ability (*P*<.001), with the PD-MCI group showing significantly lower performance in these cognitive domains compared to PD-NC and PD-SCD groups. In attention, the differences among the 3 groups approached statistical significance (*P*=.05), with the PD-MCI group showing a trend toward decreased attention. However, no statistically significant differences were observed among the 3 groups in executive function (*P*=.25), language (*P*=.83), naming (*P*=.45), and orientation (*P*=.11; Figure 2). These findings align with the disease progression pattern of PD. As a chronic neurodegenerative condition, cognitive function may gradually deteriorate with disease progression, particularly in higher-order cognitive functions such as memory, visuospatial ability, and abstract thinking. The significant decline in delayed recall and visuospatial ability observed in patients with PD-MCI reflects the typical characteristics of PD-related cognitive impairment. In contrast, language, naming, and executive functions remain relatively preserved in the early stages, which is consistent with the domain-specific pattern of cognitive impairment in PD.

### Characteristics of Kinematic Parameters in Groups of PD-NC, PD-SCD, and PD-MCI

Screening procedures identified 45 kinematic parameters meeting the established criteria, distributed across TUGst (n=25), TUGdt (n=12), and DTC (n=8) paradigms. Gait phase analysis revealed 35 parameters from walking phases, 9 from stand-to-sit transitions, and 1 from sit-to-stand transitions. Feature type distribution demonstrated a predominance of variability features (n=20), followed by pace (n=12) and axial (n=8) characteristics. This distribution pattern reflects differential impacts of cognitive decline on gait control mechanisms, with movement precision (variability) and temporal coordination (pace) emerging as critical cognitive function indicators. TUGdt paradigm analysis revealed pronounced movement differences between PD-MCI and both PD-NC and PD-SCD groups, particularly in variability, amplitude, pace, and axial domains. Notable examples include “Shank-Max Sagittal Angular Velocity R Std” (21.12 vs 28.38 vs 21.38) and “Trunk-Left Rotation Max Std” (1.94 vs 1.99 vs 1.33) in variability measures. Amplitude analysis of “Shank-Symbolic Symmetry Index” demonstrated compensatory patterns (14.27 vs 9.75 vs 12.62), suggesting potential compensatory mechanisms during SCD stages. TUGst paradigm differences primarily manifested in pace characteristics, exemplified by “Double Support” (23.16 [4.76] vs 18.55 [3.04] vs 20.87 [3.75]) and “Stance _MAXLR” (62.17 [2.75] vs 60.06 [1.95] vs 61.17 [2.09]). DTC parameter analysis revealed a concentration of differences in variability features, including “Gait Speed R.Std” (–0.97 vs –0.30 vs –0.14) and “Shank-Max Sagittal Angular Velocity R.Std” (–1.67 vs –0.33 vs –0.30), demonstrating cognitive load effects on motor performance. Cross-paradigm analysis identified consistent significant differences in specific features, such as “Step Length_ABSLR” in DTC (–0.36 vs 0.16 vs 0.14), establishing step length asymmetry as a robust cognitive function biomarker. These findings provide objective kinematic biomarkers for early cognitive state identification in PD, with TUGdt parameters demonstrating superior discriminative capacity.

Multimedia Appendix 3 results demonstrate systematic kinematic feature differences across cognitive states, primarily in movement precision (variability) and temporal coordination (pace) domains. Enhanced differences in TUGdt paradigms align with theoretical frameworks predicting motor control degradation under increased cognitive load conditions. Compensatory pattern identification (eg, preserved performance in SCD stages) suggests early-stage compensatory mechanisms maintaining motor function. However, progressive cognitive decline appears to compromise these mechanisms, resulting in significant motor deterioration in MCI stages. These findings establish objective kinematic biomarkers for early cognitive state identification and monitoring in PD, with TUGdt variability features demonstrating particular clinical translational potential.

**Figure 3:**
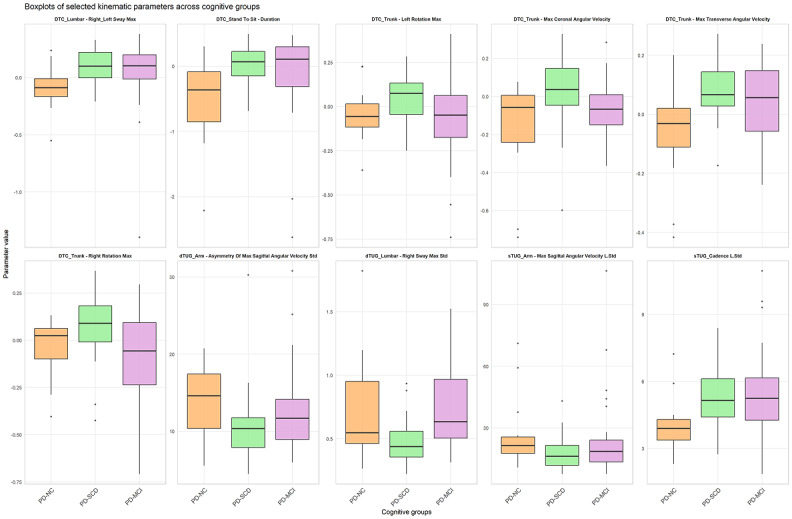
Multiple-comparison results of represented kinematic parameters between pairs of groups. Multiple-comparison results of represented kinematic parameters between pairs of groups. DTC: dual-task cost; PD-MCI: Parkinson disease-mild cognitive impairment; PD-NC: Parkinson disease-normal cognition; PD-SCD: Parkinson disease-subjective cognitive decline.

Figure 3 presents boxplots illustrating the distribution of 10 selected kinematic parameters across the PD-NC, PD-SCD, and PD-MCI cognitive groups, visually demonstrating the differences among these groups for each parameter. The box in each boxplot represents the 25th percentile (Q1, bottom line of the box), the median (central line of the box), and the 75th percentile (Q3, top line of the box). The bottom and top of the error bars (whiskers) indicate the “Minimum” (Q1-1.5×IQR) and “Maximum” (Q3+1.5×IQR), where IQR is the interquartile range. Blue dots outside this range represent outliers.

As observed from the figure, for several DTC related parameters, such as “DTC_Lumbar-Right_Left Sway Max,” “DTC_Stand To Sit-Duration,” “DTC_Trunk-Max Transverse Angular Velocity,” and “DTC_Trunk-Right Rotation Max,” the median values for the PD-NC group are generally lower than those for the PD-SCD and PD-MCI groups, indicating lower motor costs under dual-task conditions in the normal cognition group. This aligns with the theory that impaired cognitive function leads to decreased motor control ability. For instance, “DTC_Stand To Sit-Duration” shows a median of approximately –0.05 for the PD-NC group, while the medians for the PD-SCD and PD-MCI groups are significantly higher than 0.20, suggesting that patients with cognitive impairment are more affected by cognitive challenges during the stand-to-sit transition. In dTUG (dual-task TUG) -related parameters, such as “dTUG_Arm-Asymmetry Of Max Sagittal Angular Velocity SD” and “dTUG_Lumbar-Right Sway Max SD,” the median values for the PD-SCD group appear slightly lower than those for the PD-NC and PD-MCI groups. This might reflect a compensatory mechanism or different motor strategies used during the SCD stage. For sTUG (single-task TUG) related parameters, such as “sTUG_Cadence L SD,” the median for the PD-NC group (approximately 3.5) is notably lower than that for both PD-SCD and PD-MCI groups (both approximately 5.5), indicating lower gait variability in cognitively normal patients under single-task conditions, whereas patients with cognitive impairment exhibit higher gait variability. These visual differences are consistent with the statistically significant results reported in Multimedia Appendix 3, further supporting the potential of these kinematic parameters as biomarkers for distinguishing different cognitive states.

### Modeling and Interpretability

**Figure 4:**
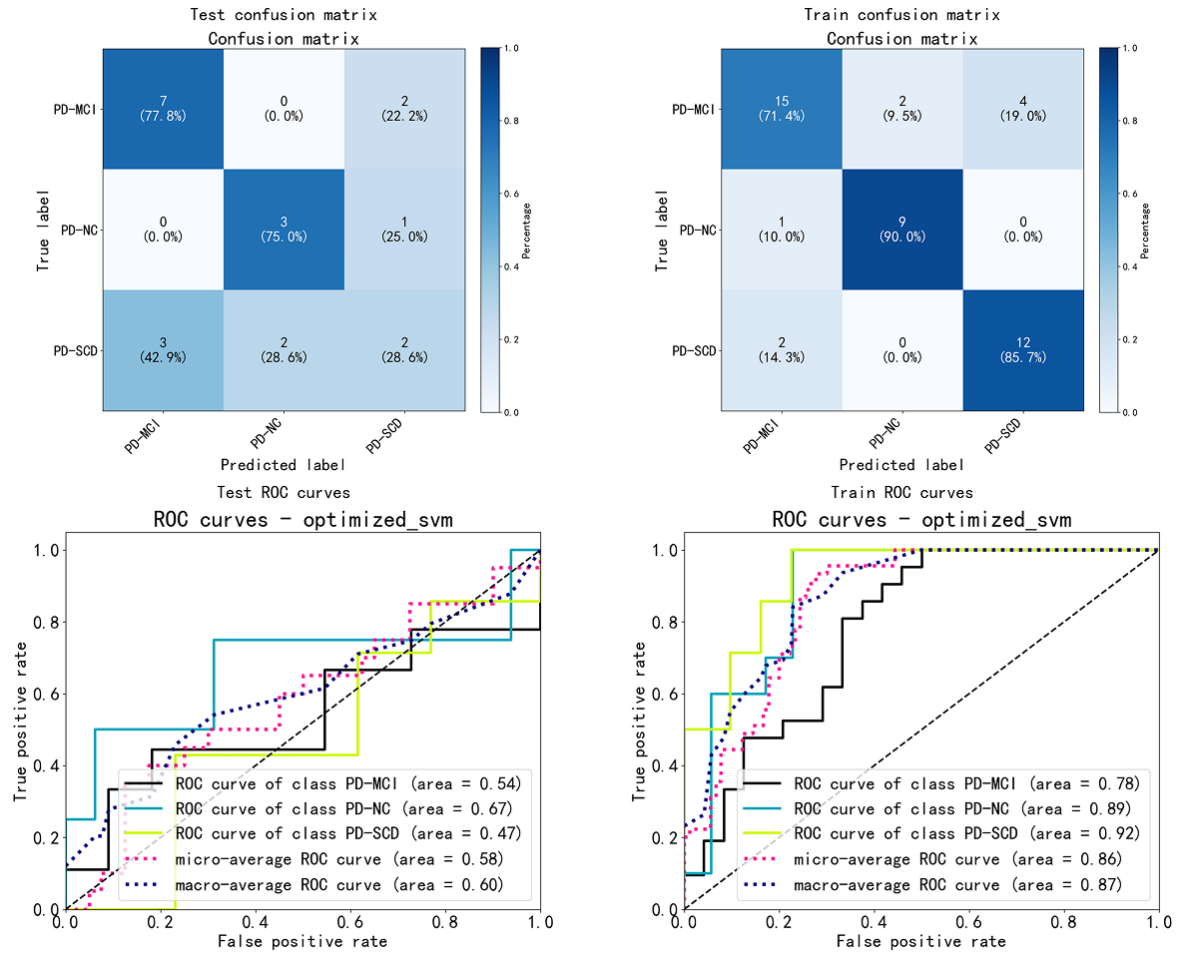
Confusion matrices and receiver operating characteristic (ROC) curves. PD-MCI: Parkinson disease-mild cognitive impairment; PD-NC: Parkinson disease-normal cognition; PD-SCD: Parkinson disease-subjective cognitive decline; ROC: receiver operating characteristic curve.

The best-performing model in the 3-class classification was the SVM model. Its classification accuracies on the training and test sets were 0.8 (0.69-0.86) and 0.6 (0.45-0.73), respectively, indicating a good performance on the training set but reduced performance on the test set (Multimedia Appendix 4). Regarding recognition capability per category on the training set, PD-MCI had a relatively higher recall (0.71), while the PD-NC and PD-SCD categories exhibited relatively higher precision, at 0.90 and 0.86, respectively. On the test set, PD-MCI maintained a relatively higher recall (0.78), while PD-NC and PD-SCD demonstrated relatively lower precision, at 0.75 and 0.29, respectively. Based on the receiver operating characteristic (ROC) curves (Figure 4), the macro-average ROC AUC on the training set was 0.87, compared to 0.60 on the test set.

**Figure 5:**
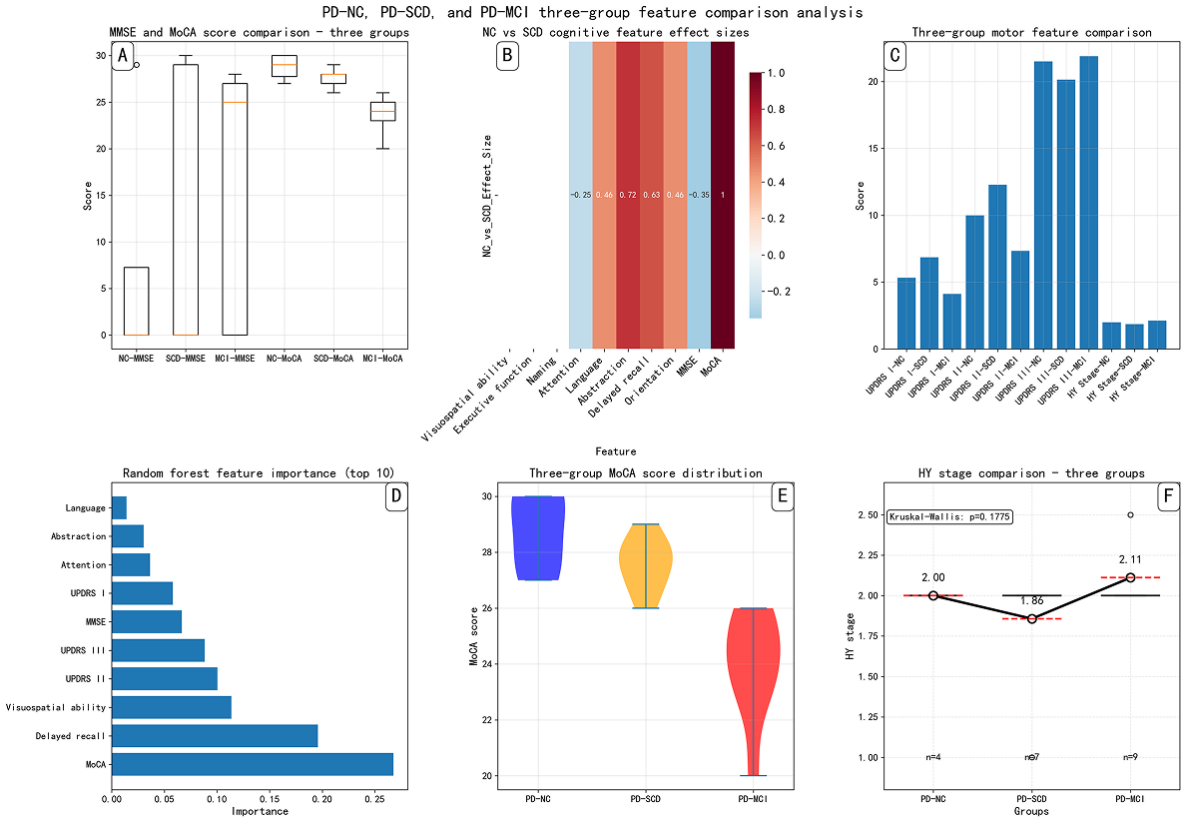
Comprehensive analysis of cognitive and motor features across Parkinson disease-normal cognition (PD-NC), Parkinson disease-subjective cognitive decline (PD-SCD), and Parkinson disease-mild cognitive impairment (PD-MCI) groups. MoCA: Montreal Cognitive Assessment; PD-MCI: Parkinson disease-mild cognitive impairment; PD-NC: Parkinson disease-normal cognition; PD-SCD: Parkinson disease-subjective cognitive decline.

Integrating participants’ cognitive levels allows for a deeper explanation of the mechanistic differences in classifying MCI, SCD, and NC (Multimedia Appendix 5, Figure 5, and Multimedia Appendix 6). Regarding the distinction between MCI and NC, cognitive level analysis revealed significant differences between the 2 groups across multiple cognitive dimensions: the MoCA total score of the MCI group was significantly lower than that of the NC group (effect size, Cohen *d*=2.672, indicating a large effect); delayed recall ability was significantly lower in the MCI group than in the NC group (Cohen *d*=1.915); visuospatial ability was significantly lower in the MCI group than in the NC group (Cohen *d*=1.354). These significant differences in objective cognitive function provide a cognitive load basis for TUG task features to effectively distinguish MCI from NC. Due to limited cognitive resources, patients with MCI exhibit significant degradation in motor performance during dual-task TUG. This cognitive-motor interaction pattern can be effectively captured by TUG features.

Regarding the distinction between SCD and NC, cognitive level analysis revealed the fundamental cause of confusion. The SCD and NC groups showed no significant difference in MoCA total score (*P*=.14). Except for abstract ability, no significant differences were found in other cognitive subdomains, with most comparisons showing small-to-medium effect sizes.

This similarity in objective cognitive function explains why TUG features struggle to distinguish SCD from NC: although patients with SCD subjectively perceive cognitive changes, their objective cognitive resources are sufficient, leading to similar performance in dual-task TUG as NC, resulting in TUG features failing to effectively distinguish these 2 groups.

Concerning the distinction between MCI and SCD, cognitive level analysis showed significant differences in objective cognitive function between the 2 groups: the MoCA total score of the MCI group was significantly lower than that of the SCD group (Cohen *d*=2.301, large effect); delayed recall ability was significantly lower in the MCI group than in the SCD group (Cohen *d*=1.423); visuospatial ability was significantly lower in the MCI group than in the SCD group (Cohen *d*=1.528). However, despite significant cognitive differences between MCI and SCD, TUG features still faced difficulties distinguishing them, with 42.9% of SCD samples misclassified as MCI.

Feature importance analysis of cognitive levels further validated the cognitive level analysis results. The MoCA total score had the highest discriminative power (*F*_2,16_=18.94; *P*<.001), followed by delayed recall (*F*_2,16_=11.70; *P*<.001). This aligns highly with the significant decline in delayed recall observed in the MCI group in the cognitive level analysis (effect size, Cohen *d*=1.915; Figure 5). The decline in delayed recall ability may directly impact patient performance in dual-task TUG, as this task requires simultaneously processing motor and cognitive demands; the efficiency of cognitive resource allocation directly affects motor performance.

**Figure 6:**
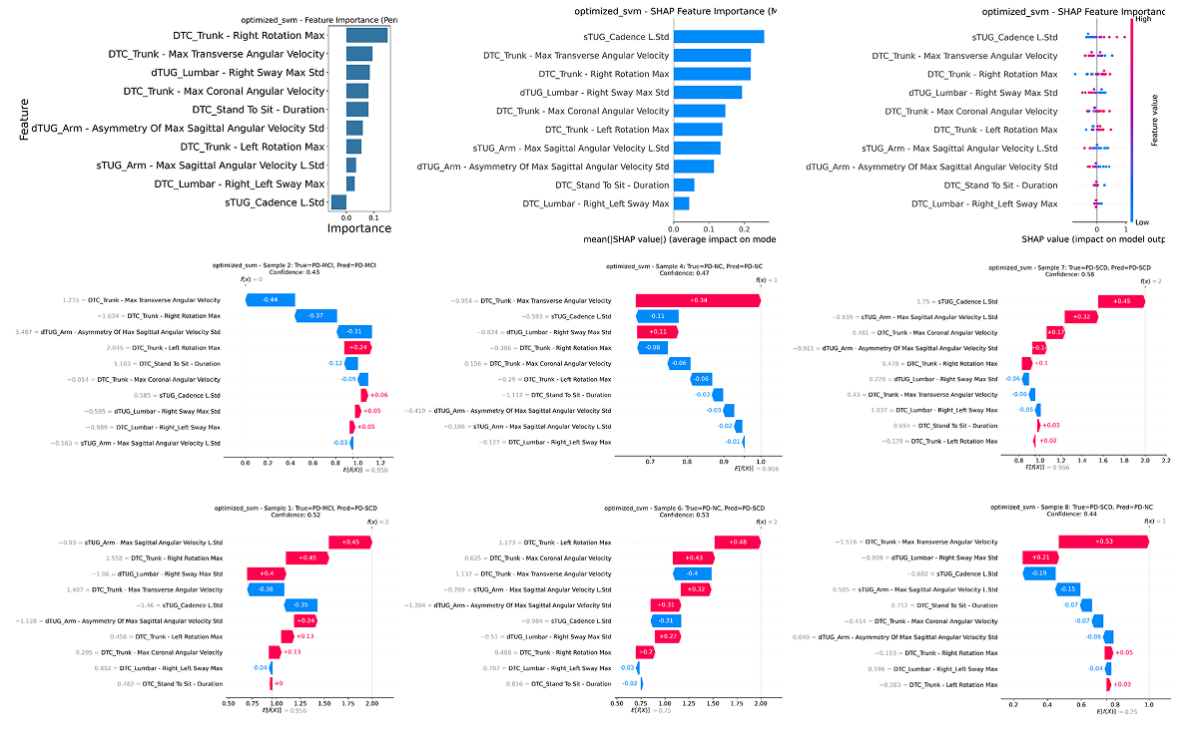
Model feature importance analysis-permutation importance and Shapley Additive Explanations (SHAP) analysis. DTC: dual-task cost; SHAP: Shapley Additive Explanations.

Furthermore, to gain a deeper understanding of the model’s decision-making mechanism, we used 2 feature importance analysis methods (Figure 6). Permutation importance analysis evaluated the impact of features on model performance by randomly shuffling feature values, revealing that DTC_Trunk-Right Rotation Max (importance value=0.145) was the most critical feature, followed by DTC_Trunk-Max Transverse Angular Velocity (0.090) and dTUG_Lumbar-Right Sway Max Std (0.080). These findings emphasize the importance of trunk motor control under dual-task conditions for distinguishing cognitive states. SHAP analysis provided 3 different perspectives on feature importance assessment. The SHAP feature importance bar plot showed that sTUG_Cadence L Std (mean [SHAP]=0.23) had the highest average impact, followed by DTC_Trunk-related features. This discrepancy with permutation importance results reflects the different mechanisms of the 2 analysis methods; permutation importance focuses on the impact of feature absence on overall model performance, while SHAP analysis focuses on the direct impact of feature value changes on model output. The SHAP feature importance summary plot further revealed the complex relationship between feature values and their impact on model output. For most DTC features, higher feature values tended to produce larger positive SHAP values, pushing the model output toward higher values, indicating that increased trunk kinematic parameters under dual-task conditions are associated with cognitive decline. This pattern supports the cognitive-motor interaction theory, suggesting that when cognitive resources are limited, individuals exhibit greater motor variability under dual-task conditions. SHAP waterfall plots provided detailed explanations at the individual sample level, demonstrating how each feature drives predictions from baseline values to final outputs. For example, in correctly classified PD-MCI samples, DTC_Trunk-Max Transverse Angular Velocity and DTC_Trunk-Right Rotation Max contributed most strongly to predictions, while correctly classified PD-NC samples exhibited relatively stable dual-task motor features. These individual-level analyses revealed the model’s decision-making mechanism in distinguishing different cognitive states, particularly the challenges faced when processing groups with similar cognitive states (such as PD-SCD and PD-NC; Multimedia Appendix 7).

## Discussion

### Principal Results

In this study, we included 65 patients with PD exhibiting various cognitive states (PD-NC: 14 cases, PD-SCD: 21 cases, and PD-MCI: 30 cases) and explored the method of using kinematic characteristics for the stratified cognitive stage classification of patients with PD by combining wearable sensors with machine learning. We first described the gait characteristics of PD-NC, PD-SCD, and PD-MCI, respectively; second, we explored the factors associated with gait kinematic parameters and cognitive status; next, we analyzed significantly different gait parameters among groups; and finally, we established a model that relies on various TUG task outcomes, which can distinguish individuals with PD-NC, PD-SCD, and PD-MCI. Our results demonstrated that kinematic features are associated with cognitive stages, patients with PD-SCD have underlying kinetic impairments similar to those with PD-MCI, and the diagnostic SVM model showed potential in differentiating cognitive impairments. The SVM model achieved classification accuracies of 0.8 (0.69-0.86) and 0.6 (0.45-0.73) on the training and test sets, respectively, with PD-MCI demonstrating the highest recall rates (0.71 on the training set, 0.78 on the test set) and macro-average ROC AUC values of 0.87 and 0.60 for the training and test sets.

Regarding the classification performance, the model demonstrated effective differentiation between MCI and NC groups due to significant differences in objective cognitive function (MoCA total score effect size, Cohen *d*=2.672), which provided a cognitive load basis for TUG task features to effectively distinguish these groups. However, the model faced significant challenges in distinguishing PD-SCD from both PD-NC and PD-MCI groups. Concerning the distinction between SCD and NC, cognitive level analysis revealed the fundamental cause of confusion. By definition, SCD refers to a subjective perception of cognitive decline, which is not necessarily aligned with the actual cognitive level. For instance, substantial cognitive impairment may occur without being perceived, whereas minor declines may be perceived as significant. Thus, it is difficult to determine which condition reflects a higher level of cognition. Consequently, NC and SCD may not exhibit clear differences in cognitive impairment; the 2 groups showed no significant difference in MoCA total score (*P*=.14). It is correspondingly challenging to distinguish between them based on motor performance. This suggests that SCD may represent a subtype with a higher risk of progressing to MCI, rather than a group that is necessarily closer to MCI in terms of cognitive function.

Regarding the distinction between SCD and MCI, cognitive level analysis showed significant differences in objective cognitive function between the 2 groups (MoCA total score effect size, Cohen *d*=2.301). However, despite significant cognitive differences between MCI and SCD, TUG features still faced difficulties distinguishing them, with 42.9% of SCD samples misclassified as MCI. This seemingly paradoxical result highlights an important mechanism of TUG task features: although MoCA assessment revealed significant cognitive differences between SCD and MCI, this difference primarily manifests in static cognitive tests. In the dynamic context of dual-task TUG, cognitive differences may be insufficient to produce noticeable variations in motor performance. Specifically, while patients with SCD had significantly higher MoCA scores than patients with MCI, their relative cognitive advantage during dual-task TUG might be counterbalanced by other factors, such as attentional allocation strategies, task priority adjustments, or motor control patterns. Furthermore, UPDRS score analysis revealed that the SCD group had the lowest UPDRS III (motor examination) score (20.143), indicating relatively better motor function in patients with SCD. This motor advantage might further compensate for the impact of cognitive differences on TUG performance. This underscores that performance in the TUG task, as a cognitive-motor dual-task paradigm, is not solely influenced by static cognitive function. Instead, it results from the combined effects of dynamic cognitive-motor interactions, attentional allocation, motor control, and other factors. When cognitive differences are counterbalanced by other factors in a dynamic task context, TUG feature-based classification models struggle to accurately distinguish different cognitive states.

Additionally, the limited sample size (n=65) after age-matching may have contributed to model performance instability, with the training set containing only 45 samples and the test set containing only 20 samples, including only 7 SCD samples in the test set, resulting in insufficient sample representativeness that hindered the model’s ability to adequately validate SCD classification performance.

### Comparison With Prior Work

#### Demographic Comparation With Other Studies

Consistent with other studies [8,20,40] patients with PD-MCI showed older age, a higher proportion of lower education, worse motor function assessed by MDS-UPDRS-III, and worse performance in global cognition and cognitive domains than the other 2 groups. Additionally, we observed a decrease across the 3 groups in various cognitive domains, including visuospatial ability, executive function, attention, language, abstract thinking, memory, and orientation. Specifically, the PD-NC group had the highest proportion of perfect scores, followed by patients with PD-SCD, with the PD-MCI group having the lowest proportion. These findings here were partially consistent with other studies, which reported that patients with PD-SCD exhibited poorer performance in attention, executive abilities, memory, and visuospatial functions compared to individuals with PD-NC [2,41-43]. Therefore, we believe that the findings of this study have strong generalizability.

#### Gait Performance Comparison Among Paradigms

In this study, we identified 45 distinct gait parameters that demonstrated significant variance across the 3 participant groups in the TUGst, TUGdt test, and DTC test. These parameters encompassed a range of dynamic attributes, including motion amplitude, pace, speed, and axial alignment, observed during the 5-meter walking, 180° turning, and sit-to-stand phases. Our findings are in line with prior cross-sectional research that has established robust correlations between gait characteristics—such as pace, rhythm, and asymmetry—and cognitive function, as measured by the TUGst or TUGdt [22,44]. A study by Kim et al [45] revealed that individuals with PD who experience cognitive impairment exhibit a marked reduction in gait speed and stride length when contrasted with those without cognitive impairment. This underscored the adverse effect that cognitive impairment could have on the mobility of patients with PD. Our research observed a decline in step length, stride length, gait speed, and swing among all 3 participant groups (PD-NC, PD-SCD, and PD-MCI) during TUGdt as opposed to the TUGst. Also, kinematic characteristics observed during the TUGdt test exhibited a more pronounced association with the ordinal cognitive stages when compared to other assessments. Specifically, Shank-Max Sagittal Angular Velocity, Trunk-Sway Max SD, Shank-Swing Speed, Shank Forward-Backward Swing Max, gait speed, and straight-walking duration (first segment of the TUGdt test or DTC) were the top 6 features associated with cognitive performance. The result here was adjusted with covariates. This suggests that the introduction of cognitive tasks during the TUGst can disrupt the execution of motor tasks, consequently diminishing the participants' walking capabilities.

#### Gait Performance Comparison Across Participant Groups

In our analysis, we observed distinct patterns in gait performance across the different participant groups, highlighting the intricate relationship between cognitive function and motor control in PD. Here is a refined presentation of our findings:

1. Gait speed and swing speed decline: a pronounced decreasing trend in gait speed and swing speed was noted as cognitive function worsened across the 3 groups. This aligns with clinical observations where individuals with PD-MCI tend to exhibit poorer gait patterns [20]. Mechanistic insights suggest that the altered neural networks and neurotransmitter deficiencies in PD are linked to both gait disturbances and cognitive impairments [19,46]. The initiation of swinging motion is a complex interplay between passive trunk movement and the precise activation of muscles, regulated by the basal ganglia [47], indicating that cognitive decline may be associated with a slower swing speed.

2. Step and stride length variations: a significant difference in step length and stride length was found between PD-MCI and PD-NC groups, with PD-MCI showing shorter lengths, which is consistent with previous research [48,49]. Interestingly, the PD-SCD group showed longer step and stride lengths, though without statistical significance, possibly indicating early compensatory mechanisms. Neuroimaging studies have consistently demonstrated elevated activation levels in the prefrontal cortex during cognitive tasks in individuals with SCD when compared to those with normal cognition [50,51]. The prefrontal cortex is known for its involvement in attention and executive functions, both of which are closely linked to performance in the TUGdt test [52,53], the enhanced functional connectivity in this region may be an underlying neural mechanism for the decline in TUGdt performance [54]. Additional studies also demonstrated the presence of compensatory neural activity in the early stages of cognitive impairment, and the cognitive strategies can compensate for the decline in gait function [55,56]. Nevertheless, it is crucial to recognize this scarcity of research on the brain function of individuals with PD-SCD. To substantiate our hypothesis and findings, there is a clear need for more extensive, large-scale investigations into brain function within this cohort.

3. Poorest gait performance in patients with PD-MCI: patients with PD-MCI showed significantly poorer gait performance than the other 2 groups. Specifically, in the TUGdt test, both the PD-NC and PD-SCD exhibited shorter walking duration, longer step length and stride length, faster walking speed, swing speed, and angular velocity than the group of PD-MCI, suggesting that individuals with PD-MCI have poorer walking ability, and this was supported by previous studies [8,57,58].

Furthermore, when comparing the PD-MCI and PD-NC groups, we noticed significantly reduced angular velocity during turning and maximum sagittal plane angular velocity during walking in PD-MCI than in PD-NC in TUGdt (Multimedia Appendix 2). PD is characterized by a reduction in the range of motion and muscle strength at the hip, knee, and ankle joints [59,60]. Consequently, those with PD-MCI might struggle with activities like turning, which demand heightened coordination and cognitive capacity [61,62]. When comparing the PD-MCI and PD-SCD groups, we observed that the PD-MCI group had longer walking duration, support phase, stance, shorter step length, stride length, walking speed, arm swing speed, and arm swing amplitude in both TUGst and TUGdt. Our results support earlier research showing that severe cognitive impairments can lead to greater motor skill deficits in gait activities [63,64].

4. Subtle gait changes in PD-SCD: our study suggests that PD-SCD individuals show subtle changes in gait, which is supported by studies demonstrating individuals with poor walking ability during the TUG test have a significantly higher risk of developing MCI compared to those with normal walking ability [48,65].

The control of trunk posture is controlled by the frontal lobe [66,67]. Individuals with PD who have abnormal posture also experience noticeable clinical symptoms of impaired attention and executive ability [68], suggesting that there may be a shared brain activity in the frontal lobe between trunk posture abnormalities and attention and executive ability.

#### Modeling and Explanation

To automatically obtain patients’ cognitive impairment level, 10 kinematic parameters were selected as predictors to construct a multiclass SVM classification model. The recall rate for PD-MCI is above 0.7, while the accuracy for the PD-SCD and PD-NC categories is relatively low. To our knowledge, this pioneering machine learning model, despite its moderate accuracy, represents the first attempt to delineate the cognitive impairment stages in patients with PD, particularly for the PD-SCD stage. Overall, motion speed–related features appeared to contribute most to distinguishing among PD-NC, PD-SCD, and PD-MCI groups. Differences across feature-importance analysis methods reflect the complexity of model decision-making. Permutation importance emphasizes the overall contribution of features, SHAP analysis focuses on the direct impact of features on model output, and waterfall plots provide detailed explanations at the individual-sample level. These complementary analysis methods collectively revealed the important role of dual-task kinematic features in cognitive state assessment, particularly DTC features related to trunk motor control, providing scientific evidence for cognitive function screening–based on kinematic parameters.

### Limitations

There are several limitations in our study. First, the MDS-UPDRS Part I 1.1 score ≥1 evaluating SCD was too simple compared with a comprehensive screening scale. However, SCD identified by MDS-UPDRS Part I 1.1 has been revealed to be associated with the development of PD-MCI, which validates the clinical significance of our measurement tool. Second, we used a short MoCA scale to assess global cognition rather than a comprehensive neuropsychological test focused on each cognitive domain. It is necessary to confirm our results with comprehensive neuropsychological tests. Next, the classification performance of the model needs to be further improved. Furthermore, a larger sample and follow-up multiple-center studies are essential to validate our cross-sectional results.

### Conclusions

Our study examines kinematic features to distinguish between 65 patients with PD, including PD-NC, PD-SCD, and PD-MCI. Forty-five gait parameters differed significantly by group in the TUGst, TUGdt tests and DTC, with TUGdt parameters showing stronger cognitive association. Patients with PD-MCI had worse gait performance than patients with PD-NC and PD-SCD. The PD-SCD group showed reduced gait speed and swing speed but longer steps and strides, and our multiclass SVM classification model with kinematic parameters achieved a recall rate above 0.7 in both training and validation datasets. This suggests patients with PD-SCD could have early kinetic signs of cognitive impairment, positioning them between PD-NC and PD-MCI, and kinematic parameters could serve as predictors to distinguish the cognitive stage of patients with PD.

## Appendix

Multimedia Appendix 1 Supplementary table kinematic features description.

Multimedia Appendix 2 Demographic and clinic characteristics among PD-NC, PD-SCD, and PD-MCI.

Multimedia Appendix 3 Table 2 Intergroup differences of kinematic parameters in PD-NC, PD-SCD, PD-MCI.

Multimedia Appendix 4 Table 3 The accuracy in train set and test set.

Multimedia Appendix 5 Cognitive features analysis - three groups comparison.

Multimedia Appendix 6 The permutation importance of selected features.

Multimedia Appendix 7 Analysis of Cognitive Level Overlap and Classification Confusion.

## Abbreviations

DTC: dual-task cost
dTUG: dual-task Timed Up and Go
HAMA: Hamilton Anxiety Scale
HAMD: Hamilton Depression Scale
H-Y: Hoehn-Yahr
MATRIX: Motor Function and Motor Symptom Quantitative Assessment System
MCI: mild cognitive impairment
MDS: Movement Disorder Society
MDS-UPDRS: Movement Disorder Society-Unified Parkinson Disease Rating Scale
MoCA: Montreal Cognitive Assessment
NC: normal cognition
OCS: ordinal cognitive stages
PD: Parkinson disease
ROC: receiver operating characteristic
RoM: range of motion
SCD: subjective cognitive decline
SHAP: Shapley Additive Explanations
sTUG: single-task Timed Up and Go
SVM: support vector machine
TUG: Timed Up and Go
TUGdt: dual-task cognition-gait timed up and go
TUGst: single-task Timed Up and Go

## Data Availability

The datasets generated or analyzed during this study are available from the corresponding author on reasonable request.
