# Supplementary material for: Gait Analysis for Identifying Normal Cognition, Subjective Cognitive Decline, and Mild Cognitive Impairment in Parkinson Disease: Diagnostic Study
Source: JMIR Mhealth Uhealth. 2026 Jun 24;14:e69273. doi: 10.2196/69273 (PMC13347079; doi:10.2196/69273)
Supplement: Multimedia Appendix 1 [file mhealth_v14i1e69273_app1.docx]

**Supplementary Table 1** Kinematic Features Description

| **Feature Name** | **Feature Description** |
| --- | --- |
| 180°Duration 1st | Duration of the first 180-degree turn during TUG test |
| 180°Duration 2nd | Duration of the second 180-degree turn during TUG test |
| Arm - Asymmetry Of Max Sagittal Angular Velocity | Asymmetry of maximum sagittal angular velocity between left and right arms |
| Arm - Asymmetry Of Max Sagittal Angular Velocity Std | Standard deviation of arm sagittal angular velocity asymmetry |
| Arm - Backward Swing Max | Maximum backward swing angle of arms during gait |
| Arm - Backward Swing Max L.Std | Standard deviation of left arm backward swing maximum |
| Arm - Backward Swing Max R.Std | Standard deviation of right arm backward swing maximum |
| Arm - Backward Swing Max Std | Standard deviation of arm backward swing maximum |
| Arm - Difference Of Max Sagittal Angular Velocity | Absolute difference of maximum sagittal angular velocity between left and right arms |
| Arm - Difference Of Max Sagittal Angular Velocity Std | Standard deviation of arm sagittal angular velocity difference |
| Arm - Forward Swing Max | Maximum forward swing angle of arms during gait |
| Arm - Forward Swing Max L.Std | Standard deviation of left arm forward swing maximum |
| Arm - Forward Swing Max R.Std | Standard deviation of right arm forward swing maximum |
| Arm - Forward Swing Max Std | Standard deviation of arm forward swing maximum |
| Arm - Forward_Backward Swing Max _ABSLR | Absolute difference between left and right arm forward/backward swing maximum |
| Arm - Forward_Backward Swing Max _MAXLR | Maximum value between left and right arm forward/backward swing |
| Arm - Forward_Backward Swing Max _MINLR | Minimum value between left and right arm forward/backward swing |
| Arm - Max Sagittal Angular Velocity | Maximum sagittal angular velocity of arms during gait |
| Arm - Max Sagittal Angular Velocity L.Std | Standard deviation of left arm maximum sagittal angular velocity |
| Arm - Max Sagittal Angular Velocity R. Std | Standard deviation of right arm maximum sagittal angular velocity |
| Arm - Max Sagittal Angular Velocity Std | Standard deviation of arm maximum sagittal angular velocity |
| Arm - Max Sagittal Angular Velocity _ABSLR | Absolute difference between left and right arm maximum sagittal angular velocity |
| Arm - Max Sagittal Angular Velocity _MAXLR | Maximum value between left and right arm sagittal angular velocity |
| Arm - Max Sagittal Angular Velocity _MINLR | Minimum value between left and right arm sagittal angular velocity |
| Arm - Swing Range | Range of arm swing motion during gait |
| Arm - Swing Range L.Std | Standard deviation of left arm swing range |
| Arm - Swing Range R.Std | Standard deviation of right arm swing range |
| Arm - Swing Range Std | Standard deviation of arm swing range |
| Arm - Swing Range _ABSLR | Absolute difference between left and right arm swing range |
| Arm - Swing Range _MAXLR | Maximum value between left and right arm swing range |
| Arm - Swing Range _MINLR | Minimum value between left and right arm swing range |
| Arm - Symbolic Symmetry Index | Symmetry index of arm movements (0% = perfect symmetry 100% = poor symmetry) |
| Arm - Symbolic Symmetry Index Std | Standard deviation of arm symbolic symmetry index |
| Cadence | Number of steps per minute during gait |
| Cadence L.Std | Standard deviation of left side cadence |
| Cadence R.Std | Standard deviation of right side cadence |
| Cadence Std | Standard deviation of cadence |
| Cadence _ABSLR | Absolute difference between left and right side cadence |
| Cadence _MAXLR | Maximum value between left and right side cadence |
| Cadence _MINLR | Minimum value between left and right side cadence |
| Coordination | Coordination index between limbs during gait |
| Coordination Std | Standard deviation of coordination index |
| Double Support | Percentage of gait cycle time when both limbs are in contact with ground |
| Double Support Std | Standard deviation of double support time |
| Duration Std | Standard deviation of trial duration |
| Effective Trial Duration | Total effective time of the TUG trial |
| Gait Cycle | Duration of one complete gait cycle |
| Gait Cycle L.Std | Standard deviation of left side gait cycle duration |
| Gait Cycle R.Std | Standard deviation of right side gait cycle duration |
| Gait Cycle Std | Standard deviation of gait cycle duration |
| Gait Cycle _ABSLR | Absolute difference between left and right side gait cycle |
| Gait Cycle _MAXLR | Maximum value between left and right side gait cycle |
| Gait Cycle _MINLR | Minimum value between left and right side gait cycle |
| Gait Speed | Average walking speed during gait |
| Gait Speed L.Std | Standard deviation of left side gait speed |
| Gait Speed R.Std | Standard deviation of right side gait speed |
| Gait Speed Std | Standard deviation of gait speed |
| Gait Speed _ABSLR | Absolute difference between left and right side gait speed |
| Gait Speed _MAXLR | Maximum value between left and right side gait speed |
| Gait Speed _MINLR | Minimum value between left and right side gait speed |
| Left Double Support | Percentage of gait cycle time when both limbs are in contact with ground (left side reference) |
| Left Double Support Std | Standard deviation of left double support time |
| Lumbar - Backward Sway Max | Maximum backward sway angle of lumbar region |
| Lumbar - Backward Sway Max Std | Standard deviation of lumbar backward sway maximum |
| Lumbar - Forward Sway Max | Maximum forward sway angle of lumbar region |
| Lumbar - Forward Sway Max Std | Standard deviation of lumbar forward sway maximum |
| Lumbar - Forward_Backward Sway Max | Range of forward and backward sway of lumbar region |
| Lumbar - Left Rotation Max | Maximum left rotation angle of lumbar region |
| Lumbar - Left Rotation Max Std | Standard deviation of lumbar left rotation maximum |
| Lumbar - Left Sway Max | Maximum left sway angle of lumbar region |
| Lumbar - Left Sway Max Std | Standard deviation of lumbar left sway maximum |
| Lumbar - Max Coronal Angular Velocity | Maximum coronal angular velocity of lumbar region |
| Lumbar - Max Coronal Angular Velocity Std | Standard deviation of lumbar maximum coronal angular velocity |
| Lumbar - Max Sagittal Angular Velocity | Maximum sagittal angular velocity of lumbar region |
| Lumbar - Max Sagittal Angular Velocity Std | Standard deviation of lumbar maximum sagittal angular velocity |
| Lumbar - Max Transverse Angular Velocity | Maximum transverse angular velocity of lumbar region |
| Lumbar - Max Transverse Angular Velocity Std | Standard deviation of lumbar maximum transverse angular velocity |
| Lumbar - Right Rotation Max | Maximum right rotation angle of lumbar region |
| Lumbar - Right Rotation Max Std | Standard deviation of lumbar right rotation maximum |
| Lumbar - Right Sway Max | Maximum right sway angle of lumbar region |
| Lumbar - Right Sway Max Std | Standard deviation of lumbar right sway maximum |
| Lumbar - Right_Left Rotation Max | Range of right and left rotation of lumbar region |
| Lumbar - Right_Left Sway Max | Range of right and left sway of lumbar region |
| Max Angular Velocity | Maximum angular velocity during turning |
| Max Angular Velocity Std | Standard deviation of maximum angular velocity |
| Mean Angular Velocity | Mean angular velocity during turning |
| Mean Angular Velocity Std | Standard deviation of mean angular velocity |
| Mean Phase Difference | Mean phase difference between limbs during gait |
| Phase Coordination Index | Phase coordination index between limbs during gait |
| Right Double Support | Percentage of gait cycle time when both limbs are in contact with ground (right side reference) |
| Right Double Support Std | Standard deviation of right double support time |
| Shank - Asymmetry Of Max Sagittal Angular Velocity | Asymmetry of maximum sagittal angular velocity between left and right shanks |
| Shank - Asymmetry Of Max Sagittal Angular Velocity Std | Standard deviation of shank sagittal angular velocity asymmetry |
| Shank - Backward Swing Max | Maximum backward swing angle of shanks during gait |
| Shank - Backward Swing Max L.Std | Standard deviation of left shank backward swing maximum |
| Shank - Backward Swing Max R.Std | Standard deviation of right shank backward swing maximum |
| Shank - Backward Swing Max Std | Standard deviation of shank backward swing maximum |
| Shank - Difference Of Max Sagittal Angular Velocity | Absolute difference of maximum sagittal angular velocity between left and right shanks |
| Shank - Difference Of Max Sagittal Angular Velocity Std | Standard deviation of shank sagittal angular velocity difference |
| Shank - Forward Swing Max | Maximum forward swing angle of shanks during gait |
| Shank - Forward Swing Max L.Std | Standard deviation of left shank forward swing maximum |
| Shank - Forward Swing Max R. Std | Standard deviation of right shank forward swing maximum |
| Shank - Forward Swing Max Std | Standard deviation of shank forward swing maximum |
| Shank - Forward_Backward Swing Max _ABSLR | Absolute difference between left and right shank forward/backward swing maximum |
| Shank - Forward_Backward Swing Max _MAXLR | Maximum value between left and right shank forward/backward swing |
| Shank - Forward_Backward Swing Max _MINLR | Minimum value between left and right shank forward/backward swing |
| Shank - Max Sagittal Angular Velocity | Maximum sagittal angular velocity of shanks during gait |
| Shank - Max Sagittal Angular Velocity L.Std | Standard deviation of left shank maximum sagittal angular velocity |
| Shank - Max Sagittal Angular Velocity R.Std | Standard deviation of right shank maximum sagittal angular velocity |
| Shank - Max Sagittal Angular Velocity Std | Standard deviation of shank maximum sagittal angular velocity |
| Shank - Max Sagittal Angular Velocity _ABSLR | Absolute difference between left and right shank maximum sagittal angular velocity |
| Shank - Max Sagittal Angular Velocity _MAXLR | Maximum value between left and right shank sagittal angular velocity |
| Shank - Max Sagittal Angular Velocity _MINLR | Minimum value between left and right shank sagittal angular velocity |
| Shank - RoM Absolute Difference | Absolute difference in range of motion between left and right shanks |
| Shank - RoM Absolute Difference Std | Standard deviation of shank range of motion absolute difference |
| Shank - RoM Asymmetry | Asymmetry of range of motion between left and right shanks |
| Shank - RoM Asymmetry Std | Standard deviation of shank range of motion asymmetry |
| Shank - Swing Speed | Angular velocity of shank swing during gait |
| Shank - Swing Speed L.Std | Standard deviation of left shank swing speed |
| Shank - Swing Speed R.Std | Standard deviation of right shank swing speed |
| Shank - Swing Speed Std | Standard deviation of shank swing speed |
| Shank - Swing Speed _ABSLR | Absolute difference between left and right shank swing speed |
| Shank - Swing Speed _MAXLR | Maximum value between left and right shank swing speed |
| Shank - Swing Speed _MINLR | Minimum value between left and right shank swing speed |
| Shank - Symbolic Symmetry Index | Symmetry index of shank movements (0% = perfect symmetry 100% = poor symmetry) |
| Shank - Symbolic Symmetry Index Std | Standard deviation of shank symbolic symmetry index |
| Sit To Stand - AVG Duration | Average duration of sit-to-stand transition |
| Sit To Stand - Duration | Duration of sit-to-stand transition |
| Sit To Stand - Trunk - Max Lean Angle | Maximum lean angle of trunk during sit-to-stand transition |
| Sit To Stand - Trunk - Max Sagittal Angular Velocity | Maximum sagittal angular velocity of trunk during sit-to-stand transition |
| Sit To Stand - Trunk - Max_Min Lean Angle | Range of maximum and minimum lean angles of trunk during sit-to-stand transition |
| Sit To Stand - Trunk - Min Lean Angle | Minimum lean angle of trunk during sit-to-stand transition |
| Stance | Percentage of gait cycle time when limb is in contact with ground |
| Stance Asymmetry | Asymmetry of stance time between left and right limbs |
| Stance Asymmetry Std | Standard deviation of stance asymmetry |
| Stance _ABSLR | Absolute difference between left and right side stance time |
| Stance _MAXLR | Maximum value between left and right side stance time |
| Stance _MINLR | Minimum value between left and right side stance time |
| Stand To Sit - Duration | Duration of stand-to-sit transition |
| Stand To Sit - Trunk - Max Lean Angle | Maximum lean angle of trunk during stand-to-sit transition |
| Stand To Sit - Trunk - Max Sagittal Angular Velocity | Maximum sagittal angular velocity of trunk during stand-to-sit transition |
| Stand To Sit - Trunk - Max_Min Lean Angle | Range of maximum and minimum lean angles of trunk during stand-to-sit transition |
| Stand To Sit - Trunk - Min Lean Angle | Minimum lean angle of trunk during stand-to-sit transition |
| Step Duration | Duration of a single step |
| Step Duration Std | Standard deviation of step duration |
| Step Length | Length of a single step |
| Step Length .Std | Standard deviation of step length |
| Step Length L.Std | Standard deviation of left side step length |
| Step Length R.Std | Standard deviation of right side step length |
| Step Length _ABSLR | Absolute difference between left and right side step length |
| Step Length _MAXLR | Maximum value between left and right side step length |
| Step Length _MINLR | Minimum value between left and right side step length |
| Steps Std | Standard deviation of number of steps |
| Straight-Walking Duration 1st | Duration of first straight walking phase |
| Straight-Walking Duration 2nd | Duration of second straight walking phase |
| Stride Length | Length of a complete stride (two consecutive steps) |
| Stride Length Asymmetry | Asymmetry of stride length between left and right sides |
| Stride Length Asymmetry Std | Standard deviation of stride length asymmetry |
| Stride Length Difference | Absolute difference in stride length between left and right sides |
| Stride Length Difference Std | Standard deviation of stride length difference |
| Stride Length L.Std | Standard deviation of left side stride length |
| Stride Length R.Std | Standard deviation of right side stride length |
| Stride Length Std | Standard deviation of stride length |
| Stride Length _ABSLR | Absolute difference between left and right side stride length |
| Stride Length _MAXLR | Maximum value between left and right side stride length |
| Stride Length _MINLR | Minimum value between left and right side stride length |
| Stride Variability | Variability of stride length across gait cycles |
| Stride Velocity Asymmetry | Asymmetry of stride velocity between left and right sides |
| Stride Velocity Asymmetry Std | Standard deviation of stride velocity asymmetry |
| Stride Velocity Difference | Absolute difference in stride velocity between left and right sides |
| Stride Velocity Difference Std | Standard deviation of stride velocity difference |
| Swing | Percentage of gait cycle time when limb is not in contact with ground |
| Swing Absolute Difference | Absolute difference in swing time between left and right limbs |
| Swing Absolute Difference Std | Standard deviation of swing absolute difference |
| Swing Asymmetry | Asymmetry of swing time between left and right limbs |
| Swing Asymmetry Std | Standard deviation of swing asymmetry |
| Swing L.Std | Standard deviation of left side swing time |
| Swing R.Std | Standard deviation of right side swing time |
| Swing Std | Standard deviation of swing time |
| Swing _ABSLR | Absolute difference between left and right side swing time |
| Swing _MAXLR | Maximum value between left and right side swing time |
| Swing _MINLR | Minimum value between left and right side swing time |
| Trial Duration | Total duration of the TUG trial |
| Trunk - Backward Sway Max | Maximum backward sway angle of trunk |
| Trunk - Backward Sway Max Std | Standard deviation of trunk backward sway maximum |
| Trunk - Forward Sway Max | Maximum forward sway angle of trunk |
| Trunk - Forward Sway Max Std | Standard deviation of trunk forward sway maximum |
| Trunk - Forward_Backward Sway Max | Range of forward and backward sway of trunk |
| Trunk - Left Rotation Max | Maximum left rotation angle of trunk |
| Trunk - Left Rotation Max Std | Standard deviation of trunk left rotation maximum |
| Trunk - Left Sway Max | Maximum left sway angle of trunk |
| Trunk - Left Sway Max Std | Standard deviation of trunk left sway maximum |
| Trunk - Max Coronal Angular Velocity | Maximum coronal angular velocity of trunk |
| Trunk - Max Coronal Angular Velocity Std | Standard deviation of trunk maximum coronal angular velocity |
| Trunk - Max Sagittal Angular Velocity | Maximum sagittal angular velocity of trunk |
| Trunk - Max Sagittal Angular Velocity Std | Standard deviation of trunk maximum sagittal angular velocity |
| Trunk - Max Transverse Angular Velocity | Maximum transverse angular velocity of trunk |
| Trunk - Max Transverse Angular Velocity Std | Standard deviation of trunk maximum transverse angular velocity |
| Trunk - Right Rotation Max | Maximum right rotation angle of trunk |
| Trunk - Right Rotation Max Std | Standard deviation of trunk right rotation maximum |
| Trunk - Right Sway Max | Maximum right sway angle of trunk |
| Trunk - Right Sway Max Std | Standard deviation of trunk right sway maximum |
| Trunk - Right_Left Rotation Max | Range of right and left rotation of trunk |
| Trunk - Right_Left Sway Max | Range of right and left sway of trunk |
| Trunk - Sway Max | Maximum sway angle of trunk |
| Trunk - Sway Max Std | Standard deviation of trunk sway maximum |
| Turn - AVG Duration | Average duration of turning phases |
| Turn - AVG Steps | Average number of steps during turning phases |
| Walk Speed | Overall walking speed during the TUG test |
