# Supplementary material for: Gait Analysis for Identifying Normal Cognition, Subjective Cognitive Decline, and Mild Cognitive Impairment in Parkinson Disease: Diagnostic Study
Source: JMIR Mhealth Uhealth. 2026 Jun 24;14:e69273. doi: 10.2196/69273 (PMC13347079; doi:10.2196/69273)
Supplement: Multimedia Appendix 2 [file mhealth_v14i1e69273_app2.docx]

**Table1** Demographic and clinic characteristics among PD-NC, PD-SCD and PD-MCI.

| **Features** | **PD-NC (n=14)** | **PD-SCD (n=20)** | **PD-MCI (n=29)** | **p value** |
| --- | --- | --- | --- | --- |
| Age, years | 60.71±7.80 | 62.30±8.34 | 64.31±7.34 | 0.342 |
| Sex = male (%) | 8 (57.1) | 9 (45.0) | 19 (65.5) | 0.362 |
| Hight (cm) | 164.79±9.01 | 162.40±6.44 | 162.62±7.13 | 0.601 |
| Length of the Thigh (cm) | 48.93±3.79 | 47.50±3.33 | 47.76±2.90 | 0.421 |
| Weight | 62.58±13.25 | 66.39±7.52 | 58.93±10.21 | 0.063 |
| Education = ≥12 years (%) | 6 (42.9) | 9 (45.0) | 6 (20.7) | 0.144 |
| Disease duration (Months) | 28.80 [17.61, 44.39] | 21.09 [8.42, 45.10] | 38.70 [27.10, 66.04] | 0.062 |
| Hoehn-Yahr stage | 2.00 [2.00, 2.00] | 2.00 [2.00, 2.00] | 2.00 [2.00, 2.00] | 0.201 |
| MDS-UPDRS I | 5.00 [4.00, 5.00] | 6.00 [3.00, 8.25] | 6.00 [4.00, 9.00] | 0.463 |
| MDS-UPDRS II | 7.77±4.87 | 9.40±4.26 | 9.14±5.15 | 0.610 |
| MDS-UPDRS III | 20.43±7.83 | 21.20±13.83 | 24.62±8.44 | 0.358 |
| UPDRS1.1 | 0.00 [0.00, 0.00] | 1.00 [1.00, 1.00] | 1.00 [0.00, 1.00] | <0.001 |
| HAMA | 5.00 [4.00, 6.50] | 8.00 [6.00, 11.00] | 8.00 [4.00, 11.00] | 0.427 |
| HAMD | 6.00 [3.00, 6.00] | 8.00 [4.00, 8.75] | 7.00 [3.50, 9.50] | 0.299 |
| MoCA | 28.00 [27.00, 29.00] | 27.00 [27.00, 28.00] | 23.00 [22.00, 25.00] | <0.001 |

**Note: MDS-UPDRS**：Movement Disorder Society Unified Parkinson’s Disease Rating Scale；**MoCA：**Montreal Cognitive Assessment；**HAMA：**Hamilton Anxiety Scale；**HAMD：**Hamilton Depression Scale. (The demographic and clinic characteristics only includes 63 participants due to the incomplete data on 2 participants​​)
