# Supplementary material for: Gait Analysis for Identifying Normal Cognition, Subjective Cognitive Decline, and Mild Cognitive Impairment in Parkinson Disease: Diagnostic Study
Source: JMIR Mhealth Uhealth. 2026 Jun 24;14:e69273. doi: 10.2196/69273 (PMC13347079; doi:10.2196/69273)
Supplement: Multimedia Appendix 3 [file mhealth_v14i1e69273_app3.docx]

**Table 2** Intergroup differences of kinematic parameters in PD-NC, PD-SCD, PD-MCI.

| **ID** | **Feature_Name** | **stage** | **type** | **Params** | **PD-NC** | **PD-SCD** | **PD-MCI** |
| --- | --- | --- | --- | --- | --- | --- | --- |
| 1 | Arm - Backward Swing Max R.Std | Walk | Variability | sTUG | 3.06[2.47, 3.77] | 3.66[2.58, 4.20] | 2.20[1.87, 2.66] |
| 2 | Arm - Backward Swing Max Std | Walk | Variability | sTUG | 2.65[2.14, 4.00] | 3.00[2.56, 3.73] | 1.99[1.74, 2.94] |
| 3 | Arm - Forward Swing Max R.Std | Walk | Variability | dTUG | 3.60[2.29, 4.36] | 3.08[2.79, 3.51] | 2.32[1.92, 3.91] |
| 4 | Arm - Swing Range L.Std | Walk | Variability | sTUG | 4.45[2.96, 6.43] | 2.41[1.75, 5.78] | 3.44[2.75, 4.62] |
| 5 | Cadence L.Std | Walk | Variability | sTUG | 3.89[3.37, 4.30] | 5.14[4.41, 6.12] | 5.24[4.26, 6.16] |
| 6 | Coordination | Walk | Asymmetry | sTUG | -0.62[-3.74, 4.07] | 1.97[-0.23, 4.07] | 3.48[1.39, 6.48] |
| 7 | Double Support | Walk | Pace | sTUG | 23.16±4.76 | 18.55±3.04 | 20.87±3.75 |
| 8 | Double Support Std | Walk | Variability | DTC | -0.29[-0.68, -0.13] | -0.21[-0.45, 0.16] | -0.10[-0.19, 0.11] |
| 9 | Gait Speed R.Std | Walk | Variability | DTC | -0.97[-1.80, -0.24] | -0.30[-0.96, 0.08] | -0.14[-0.30, 0.32] |
| 10 | Gait Speed Std | Walk | Variability | DTC | -0.57[-1.08, -0.12] | -0.36[-0.89, 0.07] | -0.10[-0.24, 0.23] |
| 11 | Left Double Support | Walk | Pace | sTUG | 23.23±4.80 | 18.60±3.01 | 20.81±3.69 |
| 12 | Lumbar - Right Sway Max Std | Walk | Variability | dTUG | 0.55[0.46, 0.95] | 0.44[0.36, 0.56] | 0.63[0.50, 0.97] |
| 13 | Lumbar - Right_Left Sway Max | Walk | Axial | DTC | -0.09[-0.17, -0.01] | 0.10[0.00, 0.22] | 0.10[-0.01, 0.20] |
| 14 | Right Double Support | Walk | Pace | sTUG | 23.05±4.77 | 18.49±3.16 | 20.94±3.85 |
| 15 | Right Double Support Std | Walk | Variability | DTC | -0.54[-1.12, -0.38] | -0.15[-0.71, 0.17] | 0.11[-0.16, 0.32] |
| 16 | Right Double Support Std | Walk | Variability | dTUG | 3.31[2.57, 4.61] | 2.63[2.13, 3.88] | 2.80[1.91, 3.35] |
| 17 | Shank - Backward Swing Max L.Std | Walk | Variability | sTUG | 1.47[1.14, 1.88] | 1.18[1.00, 1.51] | 2.05[1.41, 2.39] |
| 18 | Shank - Max Sagittal Angular Velocity R.Std | Walk | Variability | DTC | -1.67[-2.21, -0.25] | -0.33[-0.90, 0.02] | -0.30[-0.86, 0.16] |
| 19 | Shank - Max Sagittal Angular Velocity R.Std | Walk | Variability | dTUG | 28.38[23.66, 35.15] | 21.38[15.09, 25.76] | 21.12[16.39, 27.00] |
| 20 | Shank - Symbolic Symmetry Index | Walk | Asymmetry | dTUG | 14.27[11.18, 18.50] | 9.75[8.05, 12.22] | 12.62[11.22, 15.43] |
| 21 | Shank - Symbolic Symmetry Index | Walk | Asymmetry | sTUG | 11.17[9.44, 13.92] | 8.68[7.75, 10.87] | 11.56[9.70, 13.66] |
| 22 | Sit To Stand - Trunk - Max_Min Lean Angle | Sit To Stand | Axial | dTUG | 93.87[86.51, 99.52] | 84.87[79.99, 94.65] | 87.74[76.00, 94.22] |
| 23 | Stance | Walk | Pace | sTUG | 60.94[59.51, 61.79] | 58.79[57.76, 59.71] | 59.85[58.67, 61.31] |
| 24 | Stance _MAXLR | Walk | Pace | sTUG | 62.17±2.75 | 60.06±1.95 | 61.17±2.09 |
| 25 | Stance _MINLR | Walk | Pace | sTUG | 59.81±2.73 | 57.45±1.78 | 58.54±2.47 |
| 26 | Stand To Sit - Duration | Stand To Sit | Pace | DTC | -0.36[-0.85, -0.08] | 0.07[-0.15, 0.23] | 0.11[-0.31, 0.30] |
| 27 | Stand To Sit - Duration | Stand To Sit | Pace | sTUG | 1.80±0.63 | 2.45±0.68 | 2.25±0.62 |
| 28 | Stand To Sit - Trunk - Max Lean Angle | Stand To Sit | Axial | dTUG | -69.73±11.91 | -58.80±11.46 | -64.22±12.25 |
| 29 | Stand To Sit - Trunk - Max_Min Lean Angle | Stand To Sit | Axial | sTUG | -23.86[-33.45, -19.74] | -32.92[-38.06, -26.06] | -31.25[-39.99, -23.25] |
| 30 | Stand To Sit - Trunk - Max_Min Lean Angle | Stand To Sit | Axial | DTC | -0.38[-1.05, -0.16] | 0.07[-0.16, 0.20] | -0.04[-0.38, 0.13] |
| 31 | Stand To Sit - Trunk - Min Lean Angle | Stand To Sit | Axial | sTUG | -38.09±15.22 | -25.38±9.05 | -26.84±12.18 |
| 32 | Step Length _ABSLR | Walk | Amplitude | DTC | -0.36[-2.33, 0.36] | 0.16[-0.90, 0.52] | 0.14[-0.15, 0.33] |
| 33 | Straight-Walking Duration 1st | Walk | Pace | sTUG | 4.39[3.53, 5.58] | 3.81[3.48, 4.35] | 4.21[3.48, 4.86] |
| 34 | Stride Length Asymmetry Std | Walk | Variability | DTC | -0.05[-0.32, 0.12] | -0.23[-1.44, 0.57] | -0.23[-1.00, 0.22] |
| 35 | Stride Length Difference Std | Walk | Variability | DTC | 0.00[-0.39, 0.22] | -0.22[-1.23, 0.61] | -0.21[-0.77, 0.30] |
| 36 | Stride Length R.Std | Walk | Variability | DTC | -0.96[-1.72, -0.31] | -0.01[-0.82, 0.26] | -0.11[-0.56, 0.14] |
| 37 | Swing | Walk | Pace | sTUG | 39.06[38.21, 40.49] | 41.21[40.29, 42.24] | 40.15[38.69, 41.33] |
| 38 | Swing _MAXLR | Walk | Pace | sTUG | 40.19±2.73 | 42.55±1.78 | 41.46±2.47 |
| 39 | Swing _MINLR | Walk | Pace | sTUG | 37.83±2.75 | 39.94±1.95 | 38.83±2.09 |
| 40 | Trunk - Forward Sway Max | walk | Axial | DTC | -0.02[-0.06, 0.00] | 0.01[-0.04, 0.03] | -0.04[-0.08, -0.02] |
| 41 | Trunk - Left Rotation Max Std | walk | Variability | dTUG | 1.99[1.63, 2.25] | 1.33[1.08, 1.69] | 1.94[1.50, 2.74] |
| 42 | Trunk - Left Rotation Max Std | walk | Variability | DTC | 0.00[-0.61, 0.29] | 0.26[-0.02, 0.37] | -0.19[-0.53, 0.19] |
| 43 | Trunk - Max Sagittal Angular Velocity | walk | Speed | sTUG | 13.50[11.25, 17.50] | 13.50[12.00, 14.25] | 13.00[11.00, 16.00] |
| 44 | Trunk - Max Transverse Angular Velocity | walk | Speed | DTC | -0.03[-0.11, 0.02] | 0.07[0.03, 0.14] | 0.06[-0.06, 0.15] |
| 45 | Trunk - Right_Left Sway Max | Walk | Axial | dTUG | 3.74[2.96, 5.42] | 2.95[2.16, 3.44] | 4.11[3.31, 5.02] |

**Note:** ABSLR：Absolute Difference among Left Side and Right Side; MAXLR：Maximum Value among Left Side and Right Side; MINLR：Minimum Value among Left Side and Right Side; L. Std & R. Std: Standard Deviation of Left & Right Side; Max: Maximum Value of Specific Feature; 1^st^: Features Describing the First Walking Stage; AVG: Average Value.
