# Supplementary material for: Gait Analysis for Identifying Normal Cognition, Subjective Cognitive Decline, and Mild Cognitive Impairment in Parkinson Disease: Diagnostic Study
Source: JMIR Mhealth Uhealth. 2026 Jun 24;14:e69273. doi: 10.2196/69273 (PMC13347079; doi:10.2196/69273)
Supplement: Multimedia Appendix 4 [file mhealth_v14i1e69273_app4.docx]

**Table 3** The accuracy in train set and test set

| **Train** | **precision** | **recall** | **f1-score** | **support** |
| --- | --- | --- | --- | --- |
| **PD-MCI** | 0.83 | 0.71 | 0.77 | 21 |
| **PD-NC** | 0.82 | 0.90 | 0.86 | 10 |
| **PD-SCD** | 0.75 | 0.86 | 0.80 | 14 |
| **accuracy** | **0.80** [0.69, 0.86] |  |  |  |
| **macro avg** | 0.80 | 0.82 | 0.81 | 45 |
| **weighted avg** | 0.80 | 0.80 | 0.80 | 45 |
| **Test** | **precision** | **recall** | **f1-score** | **Support** |
| **PD-MCI** | 0.70 | 0.78 | 0.74 | 9 |
| **PD-NC** | 0.60 | 0.75 | 0.67 | 4 |
| **PD-SCD** | 0.40 | 0.29 | 0.33 | 7 |
| **accuracy** | **0.60** [0.45, 0.73] |  |  |  |
| **macro avg** | 0.57 | 0.60 | 0.58 | 20 |
| **weighted avg** | 0.58 | 0.60 | 0.58 | 20 |
