# Supplementary material for: Gait Analysis for Identifying Normal Cognition, Subjective Cognitive Decline, and Mild Cognitive Impairment in Parkinson Disease: Diagnostic Study
Source: JMIR Mhealth Uhealth. 2026 Jun 24;14:e69273. doi: 10.2196/69273 (PMC13347079; doi:10.2196/69273)
Supplement: Multimedia Appendix 5 [file mhealth_v14i1e69273_app5.docx]

**Table 4** Cognitive features analysis - three groups comparison

| **Feature** | **Visuospatial ability** | **Executive function** | **Naming** | **Attention** | **Language** | **Abstraction** | **Delayed recall** | **Orientation** | **MMSE** | **MoCA** |
| --- | --- | --- | --- | --- | --- | --- | --- | --- | --- | --- |
| **PD-NC_Mean** | 2.00 | 3.00 | 3.00 | 5.75 | 3.00 | 2.00 | 4.00 | 6.00 | 7.25 | 28.75 |
| **PD-NC_SD** | 0.00 | 0.00 | 0.00 | 0.50 | 0.00 | 0.00 | 1.41 | 0.00 | 14.50 | 1.50 |
| **PD-SCD_Mean** | 2.00 | 3.00 | 3.00 | 5.86 | 2.86 | 1.71 | 3.29 | 5.86 | 12.57 | 27.57 |
| **PD-SCD_SD** | 0.00 | 0.00 | 0.00 | 0.38 | 0.38 | 0.49 | 0.95 | 0.38 | 15.69 | 0.98 |
| **PD-MCI_Mean** | 1.00 | 2.67 | 2.89 | 5.67 | 2.78 | 1.22 | 2.00 | 5.78 | 14.78 | 24.00 |
| **PD-MCI_SD** | 0.87 | 0.71 | 0.33 | 0.71 | 0.44 | 0.83 | 0.87 | 0.67 | 14.04 | 1.87 |
| **Kruskal-Wallis_H** | 9.64 | 2.57 | 1.22 | 0.26 | 1.02 | 4.09 | 7.86 | 0.54 | 0.49 | 14.34 |
| **Kruskal-Wallis_p** | 0.01 | 0.28 | 0.54 | 0.88 | 0.60 | 0.13 | 0.02 | 0.76 | 0.78 | 0.00 |
| **NC_vs_SCD_t** |  |  |  | -0.40 | 0.74 | 1.14 | 1.01 | 0.74 | -0.55 | 1.60 |
| **NC_vs_SCD_p** |  |  |  | 0.70 | 0.48 | 0.28 | 0.34 | 0.48 | 0.59 | 0.14 |
| **NC_vs_SCD_Effect_Size** |  |  |  | -0.25 | 0.46 | 0.72 | 0.63 | 0.46 | -0.35 | 1.00 |
| **NC_vs_MCI_t** | 2.25 | 0.92 | 0.65 | 0.21 | 0.98 | 1.82 | 3.19 | 0.65 | -0.88 | 4.45 |
| **NC_vs_MCI_p** | 0.05 | 0.38 | 0.53 | 0.84 | 0.35 | 0.10 | 0.01 | 0.53 | 0.40 | 0.00 |
| **NC_vs_MCI_Effect_Size** | 1.35 | 0.55 | 0.39 | 0.13 | 0.59 | 1.09 | 1.91 | 0.39 | -0.53 | 2.67 |
| **SCD_vs_MCI_t** | 3.03 | 1.24 | 0.88 | 0.64 | 0.38 | 1.38 | 2.82 | 0.28 | -0.30 | 4.57 |
| **SCD_vs_MCI_p** | 0.01 | 0.24 | 0.40 | 0.53 | 0.71 | 0.19 | 0.01 | 0.78 | 0.77 | 0.00 |
| **SCD_vs_MCI_Effect_Size** | 1.53 | 0.62 | 0.44 | 0.32 | 0.19 | 0.70 | 1.42 | 0.14 | -0.15 | 2.30 |

**Note:** Several cognitive features (visuospatial ability, executive function, naming) showed zero standard deviation in the PD-NC group or PD-SCD group, indicating that normal cognition patients performed identically on these items. Due to zero within-group variance, t-statistics and effect sizes for NC vs SCD comparisons could not be calculated.
