# Supplementary material for: Gait Analysis for Identifying Normal Cognition, Subjective Cognitive Decline, and Mild Cognitive Impairment in Parkinson Disease: Diagnostic Study
Source: JMIR Mhealth Uhealth. 2026 Jun 24;14:e69273. doi: 10.2196/69273 (PMC13347079; doi:10.2196/69273)
Supplement: Multimedia Appendix 6 [file mhealth_v14i1e69273_app6.docx]

**Table 5** The permutation importance of selected features.

| Feature | Importance |
| --- | --- |
| DTC_Trunk - Right Rotation Max | 0.149 |
| DTC_Trunk - Max Transverse Angular Velocity | 0.095 |
| dTUG_Lumbar - Right Sway Max Std | 0.085 |
| DTC_Trunk - Max Coronal Angular Velocity | 0.08 |
| DTC_Stand To Sit - Duration | 0.08 |
| dTUG_Arm - Asymmetry Of Max Sagittal Angular Velocity Std | 0.059 |
| DTC_Trunk - Left Rotation Max | 0.055 |
| sTUG_Arm - Max Sagittal Angular Velocity L.Std | 0.035 |
| DTC_Lumbar - Right_Left Sway Max | 0.03 |
| sTUG_Cadence L.Std | -0.055 |
