# Supplementary material for: Gait Analysis for Identifying Normal Cognition, Subjective Cognitive Decline, and Mild Cognitive Impairment in Parkinson Disease: Diagnostic Study
Source: JMIR Mhealth Uhealth. 2026 Jun 24;14:e69273. doi: 10.2196/69273 (PMC13347079; doi:10.2196/69273)
Supplement: Multimedia Appendix 7 [file mhealth_v14i1e69273_app7.docx]

**Supplementary Figure 1** Analysis of Cognitive Level Overlap and Classification Confusion


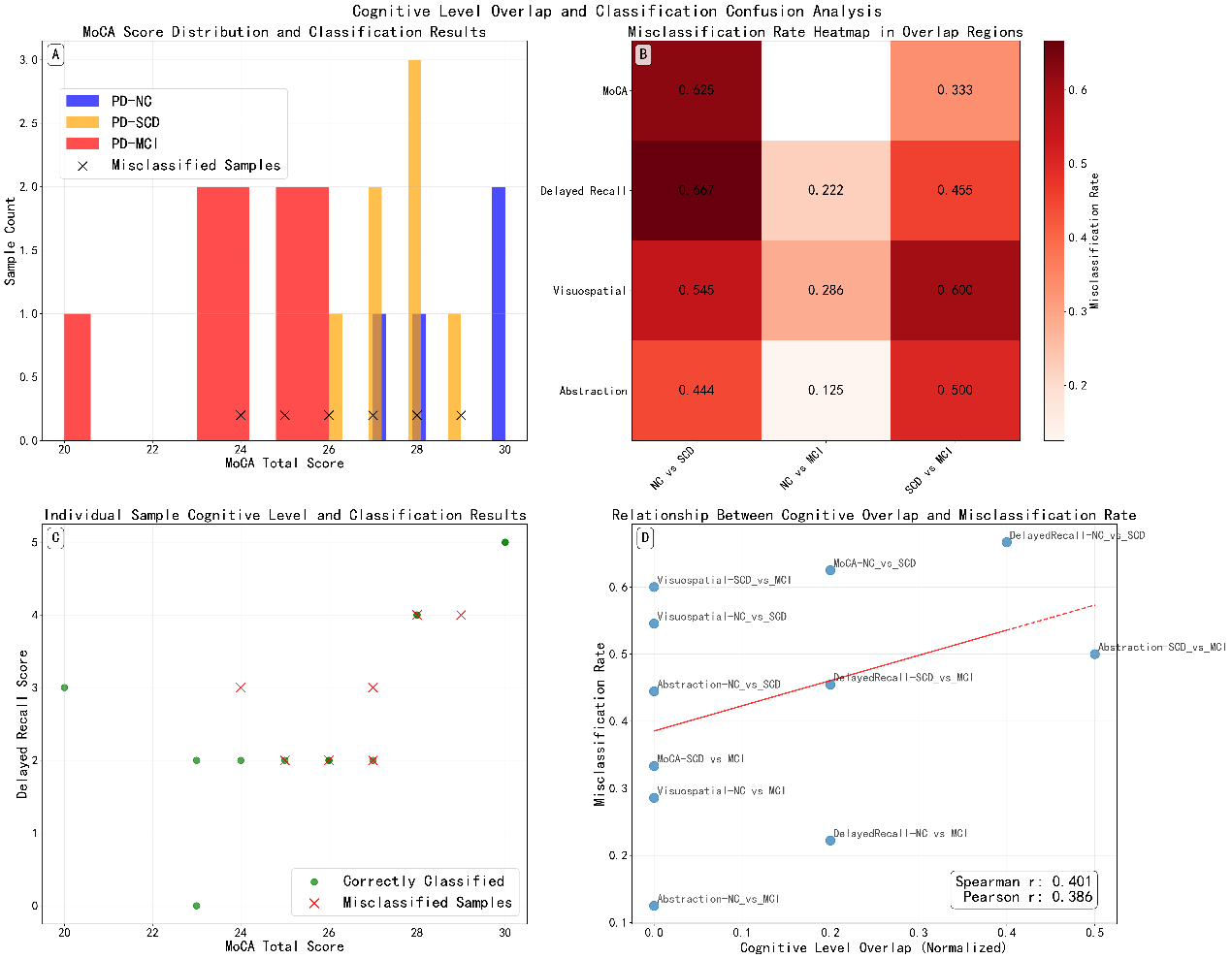


Supplementary Figure 1 shows the relationship between cognitive level overlap and classification confusion, revealing the challenges faced by machine learning models in distinguishing different cognitive states. (A) MoCA Score Distribution and Classification Results Analysis. This subplot presents the distribution of MoCA total scores across three patient groups (NC, SCD, MCI). The histogram clearly demonstrates that the NC group (blue bars) primarily concentrates within the 27-29 score range, the SCD group (orange bars) spans scores from 25-28, while the MCI group (red bars) distributes across the 20-26 range. Black crosses indicate misclassified samples, which predominantly appear in overlapping boundary regions between groups, particularly concentrated in the 26-28 score overlap interval. (B) Misclassification Rate Heatmap in Overlap Regions. This heatmap displays misclassification rates within overlap regions for different cognitive features across group comparisons. Color intensity represents misclassification rate levels, with deep red indicating high rates. Results reveal that the delayed recall feature exhibits the highest misclassification rate (66.7%) in NC vs SCD comparison, followed by the MoCA feature in NC vs SCD comparison (62.5%). Visuospatial and abstraction features also show high misclassification rates in SCD vs MCI comparison (60% and 50%, respectively). (C) Individual Sample Cognitive Level and Classification Results. This scatter plot uses MoCA total score as the x-axis and delayed recall score as the y-axis to display cognitive level distribution for each sample. Green circles represent correctly classified samples, while red crosses indicate misclassified samples. The plot reveals that misclassified samples are primarily distributed in cognitive score overlap regions, particularly in the intersection area of MoCA scores 24-28 and delayed recall scores 2-4, where correctly classified and misclassified samples are intermixed, directly reflecting the impact of cognitive level overlap on classification accuracy. (D) Relationship Between Cognitive Overlap and Misclassification Rate. This scatter plot shows the relationship between normalized cognitive level overlap (x-axis) and misclassification rate (y-axis). Each data point represents a feature-group comparison combination, with specific features and comparison combinations annotated beside points. The red dashed line shows a positive correlation trend between the two variables. The Spearman correlation coefficient is 0.401 and Pearson correlation coefficient is 0.386, indicating a moderate positive correlation between cognitive level overlap and misclassification rate.

These results validate that the primary cause of motor feature classification confusion may be the cognitive level overlap between groups, and the key findings include: (1) Cognitive level overlap shows a positive correlation with classification confusion (Spearman r=0.401); (2) MoCA total score, delayed recall, visuospatial and abstraction are primary cognitive factors contributing to classification confusion; (3) Misclassification rates within overlap regions significantly exceed overall misclassification rates, confirming that overlap regions are concentrated occurrence sites of classification errors; (4) NC vs SCD comparison exhibits the highest misclassification rate, consistent with clinical observations that SCD patients, despite subjective cognitive complaints, maintain objective cognitive function similar to the NC group. Since cognitive impairment shows no essential differences between these groups, their impact on motor function may also lack significant differences, leading to classification difficulties based on motor features; (5) SCD vs MCI comparison shows that the misclassification rates within overlap regions for visuospatial and abstraction features are 60% and 50%, respectively. This indicates that when patients from both groups exhibit overlap in specific cognitive dimensions, motor feature-based classifiers have difficulty effectively distinguishing between them.

TUG tasks include single-task and dual-task paradigms, where the dual-task requires participants to perform serial subtraction of seven while executing gait tasks, directly reflecting the complexity of cognitive-motor interactions. Kinematic features are primarily influenced by executive function, and are also affected by visuospatial ability and delayed recall [1–4].DTC (dual-task cost) parameters related to trunk features have the highest discriminative value, while visuospatial ability directly affects spatial orientation and trunk stability, and abstract ability influences task strategy formulation, both playing crucial roles in trunk motor control [5–8].Subplot B shows high misclassification rates in overlap regions for visuospatial and abstract abilities between SCD and MCI groups (60% and 50% respectively), indicating similar executive function between these groups, leading to similar motor features and ultimately classification confusion. This creates a complete logical loop: SCD indeed exhibits executive function impairment similar to MCI. When SCD and MCI show overlap in visuospatial and abstract abilities, their executive function impairment levels are similar, resulting in similar TUG task performance and indistinguishable kinematic features. This finding supports the view that “SCD is an intermediate stage between NC and MCI” rather than being merely influenced by subjective emotional factors[9–12]. The scatter plot in Subplot C further reveals that in the intersection area of MoCA scores 24-28 and delayed recall scores 2-4, samples from SCD and MCI patients show substantial overlap, with correctly classified and misclassified samples intermixed, directly illustrating the impact of cognitive level overlap on classification accuracy. Although static cognitive tests show that SCD patients significantly outperform MCI patients in MoCA total score and other aspects, during dynamic execution of dual-task TUG, these cognitive differences may be insufficient to produce significant motor performance variations. When patients from both groups exhibit overlap in specific cognitive dimensions (such as visuospatial and abstraction abilities), motor feature-based classifiers are more prone to misclassification. This supports the argument that “cognitive level overlap may be the primary cause of classification confusion.”

These findings explain why our models based on motor features face challenges in distinguishing different cognitive states among PD patients, particularly in SCD identification. Results suggest that future classification models should consider integrating multimodal features (cognitive, motor, neuroimaging, etc.) to improve identification accuracy for patients with ambiguous cognitive state boundaries. Moreover, this finding provides further clinical reference: when overall MoCA scores differ but motor features remain similar, it may indicate that impairments in certain specific cognitive domains have already reached a relatively consistent level, thereby necessitating more fine-grained domain-specific cognitive assessments.

**Reference**

1. Shim H, Kim M, Won CW. Motoric cognitive risk syndrome is associated with processing speed and executive function, but not delayed free recall memory: The Korean frailty and aging cohort study (KFACS). Arch Gerontol Geriatr 2020;87:103990. PMID:31786409

2. Weng W-H, Yang Y-R, Yeh N-C, Ku P-H, Wang P-S, Liao Y-Y, Wang R-Y. Gait performance and prefrontal cortex activation during single and dual task walking in older adults with different cognitive levels. Front Aging Neurosci 2023;15:1177082. PMID:37333460

3. Mirelman A, Bonato P, Camicioli R, Ellis TD, Giladi N, Hamilton JL, Hass CJ, Hausdorff JM, Pelosin E, Almeida QJ. Gait impairments in Parkinson’s disease. Lancet Neurol 2019 Jul;18(7):697–708. doi: 10.1016/S1474-4422(19)30044-4

4. Montero-Odasso M, Verghese J, Beauchet O, Hausdorff JM. Gait and Cognition: A Complementary Approach to Understanding Brain Function and the Risk of Falling. J Am Geriatr Soc 2012 Nov;60(11):2127–2136. PMID:23110433

5. Nieto-Escamez F, Obrero-Gaitán E, Cortés-Pérez I. Visual Dysfunction in Parkinson’s Disease. Brain Sci 2023 Aug 7;13(8):1173. PMID:37626529

6. Toots ATM, Taylor ME, Lord SR, Close JCT. Associations Between Gait Speed and Cognitive Domains in Older People with Cognitive Impairment. J Alzheimers Dis JAD 2019;71(s1):S15–S21. PMID:31256121

7. Lingo VanGilder J, Walter CS, Hengge CR, Schaefer SY. Exploring the relationship between visuospatial function and age-related deficits in motor skill transfer. Aging Clin Exp Res 2020 Aug;32(8):1451–1458. PMID:31520336

8. Amboni M, Ricciardi C, Cuoco S, Donisi L, Volzone A, Ricciardelli G, Pellecchia MT, Santangelo G, Cesarelli M, Barone P. Mild Cognitive Impairment Subtypes Are Associated With Peculiar Gait Patterns in Parkinson’s Disease. Front Aging Neurosci 2022 Mar 1;14:781480. PMID:35299943

9. Hong JY, Sunwoo MK, Chung SJ, Ham JH, Lee JE, Sohn YH, Lee PH. Subjective cognitive decline predicts future deterioration in cognitively normal patients with Parkinson’s disease. Neurobiol Aging 2014 Jul;35(7):1739–1743. doi: 10.1016/j.neurobiolaging.2013.11.017

10. Hong JY, Yun HJ, Sunwoo MK, Ham JH, Lee J-M, Sohn YH, Lee PH. Cognitive and cortical thinning patterns of subjective cognitive decline in patients with and without Parkinson’s disease. Parkinsonism Relat Disord 2014 Sep;20(9):999–1003. doi: 10.1016/j.parkreldis.2014.06.011

11. Galtier I, Nieto A, Lorenzo JN, Barroso J. Subjective cognitive decline and progression to dementia in Parkinson’s disease: a long-term follow-up study. J Neurol 2019 Mar;266(3):745–754. doi: 10.1007/s00415-019-09197-0

12. Purri R, Brennan L, Rick J, Xie SX, Deck BL, Chahine LM, Dahodwala N, Chen-Plotkin A, Duda JE, Morley JF, Akhtar RS, Trojanowski JQ, Siderowf A, Weintraub D. Subjective cognitive complaint in Parkinson disease patients with normal cognition: Canary in the coal mine? Mov Disord Off J Mov Disord Soc 2020 Sep;35(9):1618–1625. PMID:32520435
